# Metatranscriptomic analysis reveals the diversity of RNA viruses in ticks in Inner Mongolia, China

**DOI:** 10.1371/journal.pntd.0012706

**Published:** 2024-12-11

**Authors:** Si Su, Meng-Yu Cui, Li-Li Xing, Rui-Juan Gao, Lan Mu, Mei Hong, Qi-Qi Guo, Hong Ren, Jing-Feng Yu, Xiao-Yan Si, Mutu Eerde

**Affiliations:** 1 Graduate School, Inner Mongolia Medical University, Hohhot, Inner Mongolia, China; 2 Department of Pathology, Yueyang Central Hospital, Yueyang, Hunan, China; 3 Department of Public Health, The Third People’s Hospital of Anyang, Anyang, Henan, China; 4 Department of Infection Control, Second Affiliated Hospital of Inner Mongolia Medical University, Hohhot, Inner Mongolia, China; 5 School of Basic Medicine, Inner Mongolia Medical University, Hohhot, Inner Mongolia, China; 6 First Clinical College, Inner Mongolia Medical University, Hohhot, Inner Mongolia, China; 7 Department of Vector Biological and Control, Inner Mongolia Center for Disease Control and Prevention, Hohhot, Inner Mongolia, China; 8 Medical Innovation Center for Nationalities, Inner Mongolia Medical University, Hohhot, Inner Mongolia, China; Solena Ag, UNITED STATES OF AMERICA

## Abstract

**Background:**

Ticks are widely distributed throughout China and are the second most prevalent pathogen vectors in the world, following only mosquitoes. Tick bites can lead to Lyme disease, forest encephalitis, and other illnesses that may result in death under severe circumstances. Materials and methods: Ticks collected from March 2021 to May 2023 were pooled and used in metatranscriptomic analyses to gain insight into the diversity and distribution of tick-borne viruses in Inner Mongolia. Next-generation sequencing (NGS) outcomes were validated, and viral prevalence across distinct tick species was determined through the application of polymerase chain reaction (PCR) paired with Sanger sequencing.

**Results:**

A total of 20 RNA viruses belonging to at least 8 families, including *Chuviridae*, *Flaviviridae*, *Solemoviridae*, *Nairoviridae*, *Partitiviridae*, *Phenuiviridae*, *Rhabdoviridae*, and *Totiviridae*, and to unclassified families were identified by NGS. Five of the identified RNA viruses (Nuomin virus, Yezo virus, tick-borne encephalitis virus, Alongshan virus, and Beiji nairovirus) are considered human pathogens. A potential human pathogen, Mukawa virus, was also among the identified viruses. *Ixodes persulcatus* carried a significantly greater number of viral species than did *Dermacentor nuttalli*, *Hyalomma marginatum*, and *Haemaphysalis concinna*. The prevalence of coinfection with multiple viruses differed in *I*. *persulcatus* from Hinggan League and Hulun Buir, and Beiji nairovirus was the codominant virus species.

**Conclusions:**

There is a remarkable diversity of RNA viruses harboured by ticks in Inner Mongolia, with variations observed in the distribution of these tick-borne viruses across different regions and tick hosts.

## Author summary

Ticks are important vectors of zoonotic diseases and play a major role in the circulation and transmission of such diseases. In this study, ticks collected from March 2021 to May 2023 were pooled and subjected to metatranscriptomic analysis to gain insight into the diversity and distribution of tick-borne viruses in Inner Mongolia. Among the identified RNA viruses, 6 are recognized as human pathogens or potential human pathogens; these are Nuomin virus, Yezo virus, tick-borne encephalitis virus, Alongshan virus, Beiji nairovirus, and Mukawa phlebovirus. This study revealed significant diversity among RNA viruses transported by ticks in Inner Mongolia as well as variations in their geographic distribution. To effectively detect alterations in pathogen distribution, ongoing surveillance is urgently needed.

## Background

Ticks, considered the second most important vector of human disease worldwide, are the primary vectors of hundreds of disease-causing pathogens, including viruses, bacteria, protozoa, and helminths [1]. Their capacity to parasitize multiple hosts, their extensive geographical distribution, and their prolonged life cycles collectively support their pivotal role in the transmission of various diseases [2]. Several tick-borne viruses (TBVs) are associated with serious diseases in both humans and animals. Notable examples include tick-borne encephalitis virus (TBEV) [3], Nairobi sheep disease virus (NSDV) [4], Crimean-Congo haemorrhagic fever virus (CCHFV) [5,6] and severe fever with thrombocytopenia syndrome virus (SFTSV) [7]. In addition, several new viruses, such as Alongshan virus (ALSV), Songling virus (SGLV) [8], and Jingmen virus (JMTV), have emerged in the past decade [9]. Owing to the absence of effective vaccines and reliable clinical diagnostic measures, these diseases pose a constant threat to human health.

Most tick-borne viruses are RNA viruses [10]. The genomes of these viruses frequently undergo genetic mutations during replication, facilitating their spread to new habitats and hosts [11,12]. Next-generation sequencing (NGS) technology has been used extensively in tick virology research [13–16]. Recent studies using next-generation sequencing (NGS) have revealed hundreds of new tick-associated RNA viruses, greatly enhancing our understanding of the tick virome [15,17–21]. Several important culturable tick-borne viruses, such as Dadong virus [22] and Antu virus [23], have also been detected through NGS, highlighting the role of NGS in expanding the knowledge of tick virus diversity and disease transmission potential.

The Inner Mongolia region, which is long and narrow from east to west, covers an area of 1,183,000 square kilometres and contains highly diverse ecological environments. Prior investigations of tick-borne viruses in this region revealed the presence of several viruses that pose significant threats to human health, including ALSV, TBEV, SGLV and CCHFV [24]. More recently, research utilizing metatranscriptomic approaches has revealed the presence of additional tick-borne viruses, such as SZW tick virus (STV), Alxa tick phlebovirus (ATPV), and Alxa tick rhabdovirus (ATRV) [25]. These findings highlight the diversity and significance of tick-borne viruses in this area. Considering the vast expanse and the ecological complexity of Inner Mongolia, it is imperative to maintain extensive and ongoing surveillance of tick-borne viruses in the region.

In this study, we collected four tick species from three distinct ecosystems—forest, desert, and grassland—across nine sampling sites in Inner Mongolia. Metagenomic analyses and PCR were conducted to investigate the diversity of the RNA viruses carried by these ticks. Our research aims to enhance the understanding of RNA virus diversity in ticks within Inner Mongolia and to provide critical data to inform the prevention and control of tick-borne viral diseases in this area.

## Materials and methods

### Ethics statement

The collection of ticks from the body surfaces of cattle, sheep and goats in this study was verbally approved by the owners of the animals and was performed in strict accordance with the National Guidelines for Experimental Animal Welfare of China (2006–398). In addition, this study was reviewed and approved by the Medical Ethics Committee of Inner Mongolia Medical University (No. YKD202302084).

### Sample collection and identification

**Fig 1:**
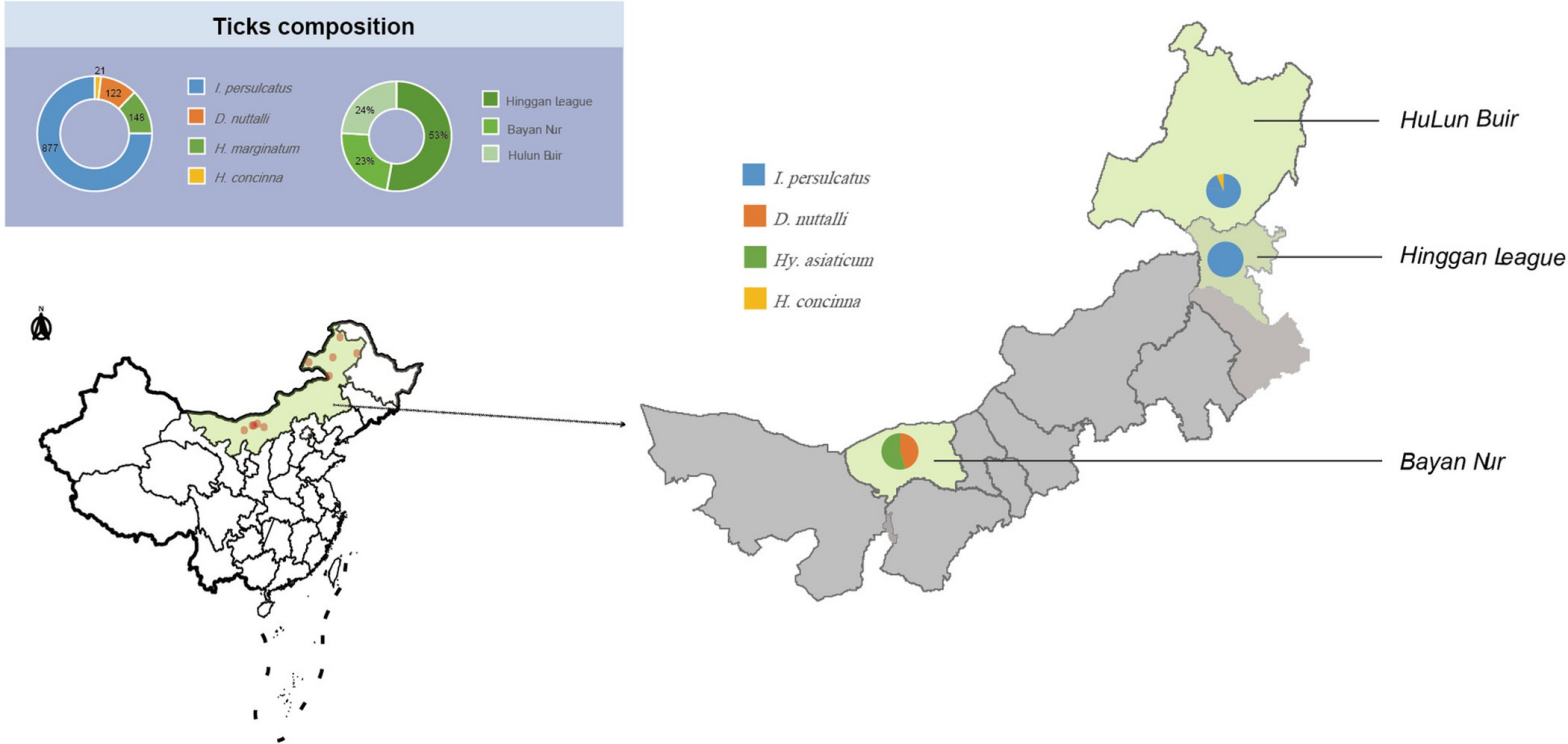
Map showing tick collection sites in Inner Mongolia, China. The map was created using the ArcGIS software program. Map source: Xinliang Xu, Multiyear administrative division boundary data of Chinese cities and municipalities, Resource and Environmental Science Data Registration and Publication System (http://www.resdc.cn/DOI, DOI: 10.12078/2023010102).

From March 2021 to May 2023, ticks of the species *Dermacentor nuttalli*, *Ixodes persulcatus*, *Hyalomma marginatum*, and *Haemaphysalis concinna* were collected from nine locations across Hulun Buir, Hinggan League, and Bayan Nur in Inner Mongolia, China (Fig 1 and Table 1). Adult ticks collected from cattle, sheep and vegetation were grouped and pooled on the basis of species and sampling location (S1 Table). The developmental stage and the species of the collected animals were initially identified on the basis of morphological characteristics by trained experts via stereomicroscopy, followed by confirmation through molecular biological methods utilizing PCR assays of mitochondrial 16S ribosomal RNA [26]. The collected samples were transported to the laboratory in ventilated bottles at low temperatures, and the ticks were then stored at -80°C for RNA library construction and subsequent analysis.

**Table 1:** Tick sampling information.

Region	Coordinates		Ecosystem and climate	Ticks (n)	Source	Species/number collected
	Longitude	Latitude				
Hinggan League	120°6’	47°3’	Forest ecosystem, humid or semihumid climate, dominated by coniferous forests, with a rich variety of vegetation	617	Forest shrub	*I*. *persulcatus/*617
	109°36’	40°55’				
Bayan Nur	107°43’	41°7’	Desert ecosystem, arid climate, sparse vegetation, low rainfall	270	Goats/sheep	*D*. *nuttalli*/122
	107°59’	41°7’				
	109°36’	40°55’				
	107°43’	41°7’			Goats	*H*. *marginatum/*148
	107°59’	41°7’				
	106°24’	40°33’				
	108°32’	41°19’				
Hulun Buir	121°51’	51°41’	Forest ecosystem, humid or semihumid climate, relatively high precipitation, relatively diverse vegetation	281	Forest shrub	*I*. *persulcatus/*260
	120°42’	49°17’	Grassland ecosystem, semihumid and semiarid climate, grasses of the family *Gramineae*, low annual precipitation			
	124°35’	49°45’	Forest ecosystem, humid or semihumid climate, relatively high precipitation, relatively diverse vegetation		Cattle/dogs	*H*. *concinna/*21

### RNA library construction and sequencing

Prior to nucleic acid extraction, each group of samples was thoroughly cleaned to eliminate potential impurities and pathogens present on their surfaces. After soaking in 75% ethanol (w/v) for 3 min, the ticks were washed three times with phosphate-buffered saline (PBS, pH = 7.4) and dried on clean filter paper. The samples in each group were ground in a mortar at a low temperature, maintained in liquid nitrogen and placed in a 1.5-ml centrifuge tube. Total RNA was extracted from the samples using a TransZol Up Plus RNA Kit (TransGen Biotech). The quality of the extracted RNA was determined using a 2100 Bioanalyzer (Agilent) and quantified via an ND-2000 (NanoDrop Technologies). High-quality RNA samples (OD260/280 = 1.8–2.2, OD260/230 ≥ 2.0) were used to construct a sequencing library.

Metatranscriptome libraries were prepared from 5 μg of total RNA according to the instructions provided with the RNA Sample Preparation Kit from Illumina (San Diego, CA). Briefly, rRNA was removed using rRNA Removal Kits (Epicenter) and fragmented in fragmentation buffer. cDNA synthesis, end repair, A-base addition and ligation of the Illumina-indexed adapters were performed according to Illumina’s protocol. The libraries were then size-selected for cDNA target fragments 200–300 bp in size by electrophoresis on 2% low-range ultra agarose, followed by PCR amplification using Phusion DNA polymerase (NEB) for 15 PCR cycles. Metatranscriptome sequencing was performed by Shanghai Biozeron Biotechnology Co., Ltd. (Shanghai, China). The samples were sequenced on an NGS platform using the Illumina NovaSeq 6000 in paired-end 150 bp (PE150) mode.

### Read quality control and mapping

For each library, sequencing quality was assessed using FastQC v0.11.9 [27], followed by quality control using Trimmomatic 0.39 to remove adapters and low-quality sequences [28]. The clean reads were subsequently *de novo* assembled into contigs using Megahit v1.2.9 [29]. For the assembled genome, Diamond blastx was used to align the assembled contigs against the RdRp-scan 0.90 database to obtain information on RNA viruses, with parameters set to "more sensitive" and a threshold of 1E-5 [30]. Further confirmation of the obtained information was conducted through online BLASTn analysis. Unless otherwise specified, all parameters were set to their default values.

### Phylogenetic analysis

To elucidate the evolutionary positions of the viruses found in ticks in Inner Mongolia, a phylogenetic analysis of the amino acid sequences of the viral RdRp proteins was performed. Reference sequences were downloaded from the NCBI database containing ICTV VMR MSL38 v3 virus classification information. For the virus sequences of interest, SeqProcessor v0.1.2 (https://pypi.org/project/seqprocessor/) was first used for sequence name modification, followed by multiple sequence alignment using MAFFT v7.450 software [31]. The phylogenetic trees were constructed using IQ-TREE v1.6.12 software [32], with the optimal substitution model selected by ModelFinder based on the Bayesian information criterion. Details regarding the best-fitting model are provided in the supplementary file (S1 File). The phylogenetic trees were visualized using Chiplot (www.chiplot.online) [33], with the roots determined by the midpoint method, providing an approximate overview of the relationships among the sequences.

### cDNA preparation and PCR for virus screening

Viral RNA was extracted using a TransZol Up Plus RNA Kit (TransGen Biotech, China), and cDNA was synthesized using One-Step gDNA Removal and cDNA Synthesis SuperMix (TransGen Biotech, China). To verify the results of NGS and determine the percentage positivity of each species for viruses of interest, oligo7 was used to design specific primers for RT–PCR. cDNA amplification for virus screening was conducted via PCR using PCR Master Mix (Tiangen, China). The amplified PCR products were separated on 1.5% agarose gels and purified for Sanger sequencing using an agarose gel purification kit.

### Statistical analysis

The viral infection rates of ticks were calculated using PooledInfRate software, version 4.0 (a Microsoft Office Excel Add-In designed by Brad J. Biggerstaff to compute prevalence estimates from pooled samples, Centers for Disease Control and Prevention, Fort Collins, CO, USA, 2024). The data were analysed using SPSS version 21.0. Differences in the percentage of infection rates of viruses in ticks were evaluated via Fisher’s exact test, with a significance threshold of p < 0.05.

## Results

### Tick collection and identification

A total of 1,168 adult ticks were collected and identified from Hulun Buir (n = 281), Bayan Nur (n = 270), and Hinggan League (n = 637); the collected ticks included 897 *I*. *persulcatus*, 122 *D*. *nuttalli*, 148 *H*. *marginatum*, and 21 *H*. *concinna* (Fig 1 and Table 1). We divided 130 sample pools into five groups according to species and geographical origin, each representing a unique tick species from a specific region: *I*. *persulcatus* from the Hinggan League region (library D), *D*. *nuttalli* from the Bayan Nur region (library B), *H*. *marginatum* from the Bayan Nur region (library E), *H*. *concinna* collected in Hulun Buir (library G) and *I*. *persulcatus* collected in Hulun Buir (library K).

### Identification of RNA viruses

Five RNA libraries were constructed and sequenced, resulting in 71.1 gigabases (GB) of high-quality, clean data comprising 480 million nonribosomal RNA (non-rRNA) reads. *De novo* assembly of these data yielded 405 distinct viral sequences from approximately 980,000 viral reads, constituting 0.2% of the total non-rRNA reads. The percentage of viral reads ranged from 0.006% (library G) to 44.0% (library K) of the total nonhost (no host rate) reads. After further confirmation via online BLASTn analysis, the viral contigs were ultimately annotated to at least 8 viral families and 20 distinct viral species, as detailed in Table 2. Notably, 5 families of vertebrate-infecting viruses were identified, namely, *Chuviridae*, *Flaviviridae*, *Nairoviridae*, *Phenuiviridae*, and *Rhabdoviridae*. These families included 6 viruses potentially associated with human and livestock diseases, namely, Nuomin virus (NUMV), Yezo virus (YZV), TBEV, ALSV, Mukawa phlebovirus (MKWV), and Beiji nariovirus (BJNV).

In a comparative analysis of the five RNA libraries, library D presented the greatest diversity, with 13 detected viruses, followed by library K, with 11 viruses, and libraries B and E, each of which contained only 3 viruses (S2 Table). Library G contained only 2 viruses. Ten viruses were shared between libraries K and D; these represented several viral families: *Chuviridae* (Nuomin virus), *Flaviviridae* (tick-borne encephalitis virus, Alongshan virus), *Nairoviridae* (Beiji nairovirus), *Phenuiviridae* (Mukawa phlebovirus, Onega tick phlebovirus, Sara tick-borne phlebovirus), *Rhabdoviridae* (Tahe rhabdovirus 2, Tahe rhabdovirus 3) and *Solemoviridae* (*Ixodes scapularis*-associated virus 1).

Among the 20 viruses identified, 14 were harboured by *I*. *persulcatus* ticks, and 3 were carried by *D*. *nuttalli* ticks and *H*. *marginatum* ticks. *H*. *concinna* ticks carried the least variety of RNA viruses, with only 2 viruses detected. Notably, the majority of the viruses are species-specific.

**Table 2:** Viruses identified in the present study.

Classification	Virus (abbreviation)	Contig length (bp)	Closest relative (accession no)	Closest relative (% nt identity)	No.unique contigs
*Bunyavirales*					
unclassified *Bunyavirales*	Volzhskoe tick virus (VSTV)	1,328	Volzhskoe tick virus strain Hun/Hy_marginatum/08-2021	87.3	1
*Flaviviridae*					
*Pestivirus-like*	Bole tick virus 4 (BLTV4)	7,389–16,653	Bole tick virus 4 strain Iftin/H.dromedarii/2018	93.2–97.5	2
*Flavivirus*	Tick-borne encephalitis virus (TBEV)	11,037–11,083	Tick-borne encephalitis virus strain TSA-18	98.0–99.6	2
*Jingmenvirus*	Alongshan virus (ALSV)	1,446–2,345	Alongshan virus strain NE-TH4	95.8–99.5	4
*Nairoviridae*					
*Norwavirus*	Beiji nariovirus (BJNV)	1,941–13,243	Beiji nariovirus strain NE-TH3	98.4–99.8	4
*Orthonairovirus*	Yezo virus (YZV)	1,620–12,118	Yezo virus isolate TIGMIC_3	98.6–99.0	3
*Chuviridae*					
*Mivirus*	Nuomin virus (NUMV)	8,780–11,041	Nuomin virus strain SL4	99.8–99.9	2
*Nigecruvirus*	Taiga tick nigecruvirus (TGTNV)	11,447	Taiga tick nigecruvirus isolate HLJ-IP-18	98.0	1
*Mivirus*	Bole Tick Virus 3 (BLTV3)	1,293	Bole Tick Virus 3 isolate GSC346MV	98.6	1
*Partitiviridae*					
*Deltapartitivirus-like*	Jilin partiti-like virus 1 (JLPV1)	1,714	Jilin partiti-like virus 1 strain MDJ2	99.7	1
*Phenuiviridae*					
*Phlebovirus*	Mukawa virus (MKWV)	1,960–6,486	Mukawa virus strain NE-TH3	89.1–98.8	5
*lxovirus*	Onega tick phlebovirus (OTPV)	6,662–1,921	Onega tick phlebovirus strain NE-SL3	99.6–100.0	3
*lxovirus*	Sara tick phlebovirus (STPV)	6,679–2,470	Sara tick phlebovirus strain NE-SL4	99.6–100.0	4
*Rhabdoviridae*					
*Alphanemrhavirus-like*	Tahe rhabdovirus 1 (THRV1)	11,362	Tahe rhabdovirus 1 strain NE-SL1	99.6	1
*Alphanemrhavirus-like*	Tahe rhabdovirus 2 (THRV2)	11,488–11,567	Tahe rhabdovirus 2 strain NE-TH4	98.7–99.2	2
*Alphanemrhavirus-like*	Tahe rhabdovirus 3 (THRV3)	5,324–10,365	Tahe rhabdovirus 3 strain NE-TH3	99.7–99.9	2
*Solemoviridae*					
*Sobemo-like*	Xinjiang tick associated virus 1 (XTAV1)	2,588	Xinjiang tick associated virus 1 isolate NM-DS-18	98.9	1
*Sobemo-like*	Ixodes scapularis associated virus 1 (ISAV1)	2,603–2,654	Ixodes scapularis associated virus 1 strain YC3	98.5–99.2	2
*Sobemo-like*	Jilin luteo-like virus 2 (JLLV2)	1,120	Jilin luteo-like virus 2 strain DH3	98.9	1
*Totiviridae*					
unclassified *Totiviridae*	Totiviridae sp.	5,706–9,022	Totiviridae sp. isolate TIGMIC_1	96.9–98.6	2

### Virus composition and evolution

**Fig 2:**
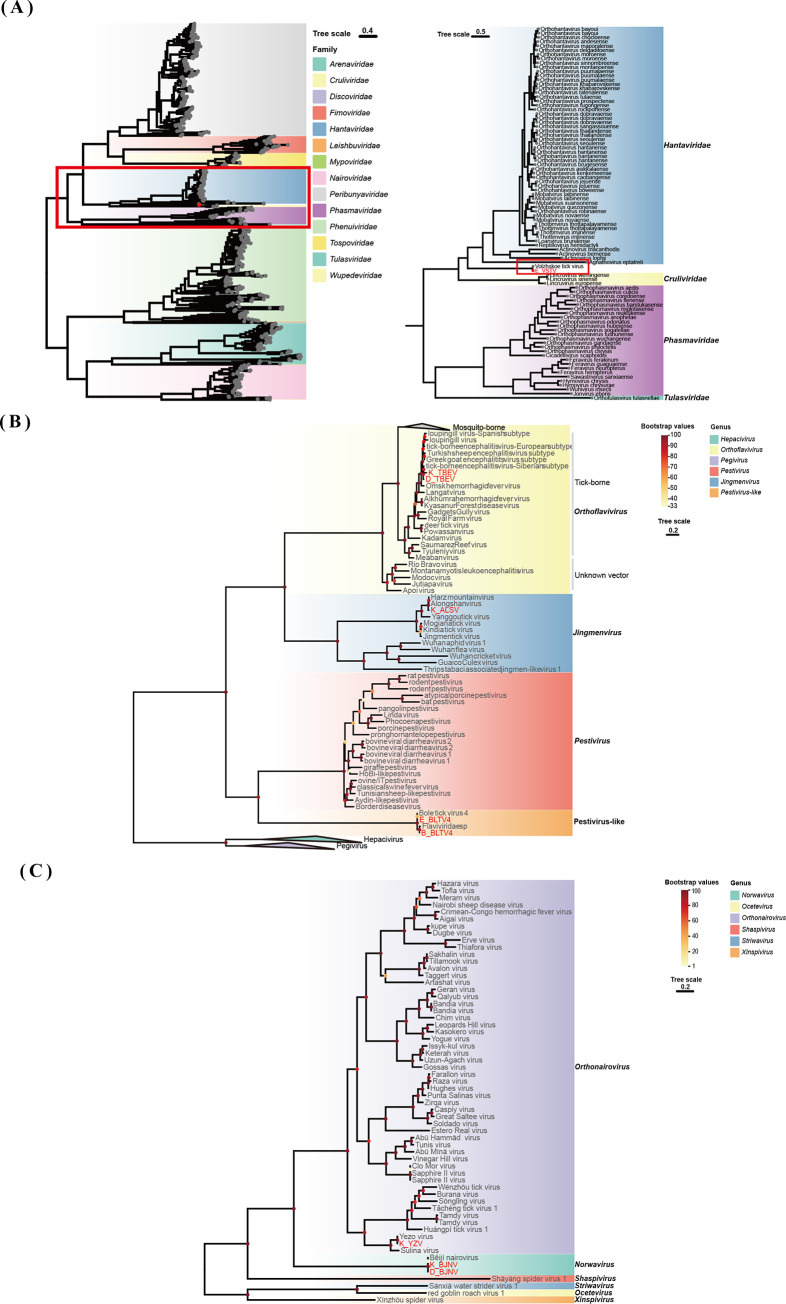
Phylogenetic analysis of novel strains of *Bunyavirales*, *Flaviviridae* and *Nairoviridae*. ML tree of representative viruses based on the RdRp genes. (A) ML tree of *Bunyavirales* viruses. (B) ML tree of viruses of *Flaviviridae*. (C) ML tree of viruses of *Nairoviridae*. The viruses newly identified in this study and previously identified viruses are shown in red and black, respectively. The best-fit amino acid substitution model for each phylogenetic tree was LG + F + R10 for *Bunyavirales*, LG + F + R7 for *Flaviviridae*, and LG + F + G4 for *Nairoviridae*.

**Fig 3:**
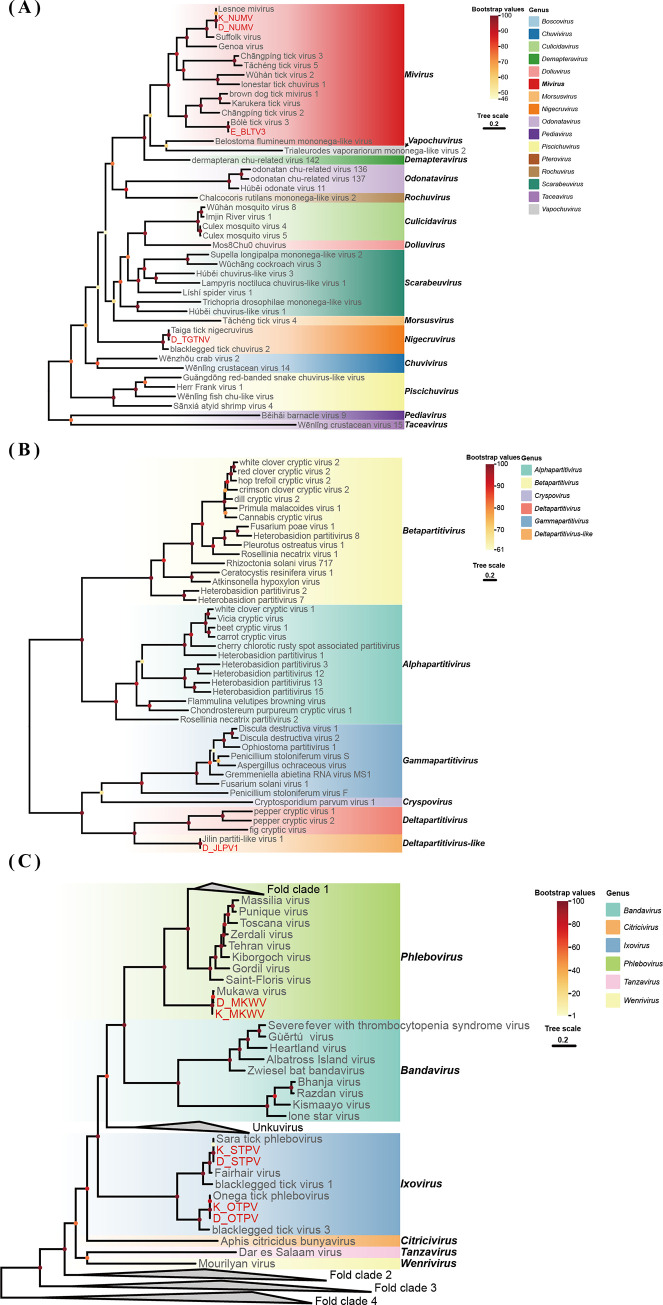
Phylogenetic analysis of the novel strains of *Chuviridae*, *Partitiviridae* and *Phenuiviridae*. ML tree of representative viruses based on the RdRp genes. (A) ML tree of *Chuviridae* viruses. (B) ML tree of *Partitiviridae* viruses. (C) ML tree of *Phenuiviridae* viruses (**Fold clade 1** includes several members of the genus *Phlebovirus*. **Fold clade 2** includes *Phasivirus*, *Beidivirus*, *Hudivirus*, *Pidchovirus*, *Hudovirus*, *Tenuivirus*, *Mechlorovirus*, and *Horwuvirus*. **Fold clade 3** includes *Goukovirus* and *Mobuvirus*. **Fold clade 4** includes *Coguvirus*, *Laulavirus*, *Lentinuvirus*, *Entovirus*, and *Rubodvirus*). The viruses newly identified in this study and previously identified viruses are shown in red and black, respectively. The best-fit amino acid substitution model for each phylogenetic tree was LG + I + G4 for *Chuviridae*, LG + F + I + G4 for *Partitiviridae*, and LG + F + R10 for *Phenuiviridae*.

**Fig 4:**
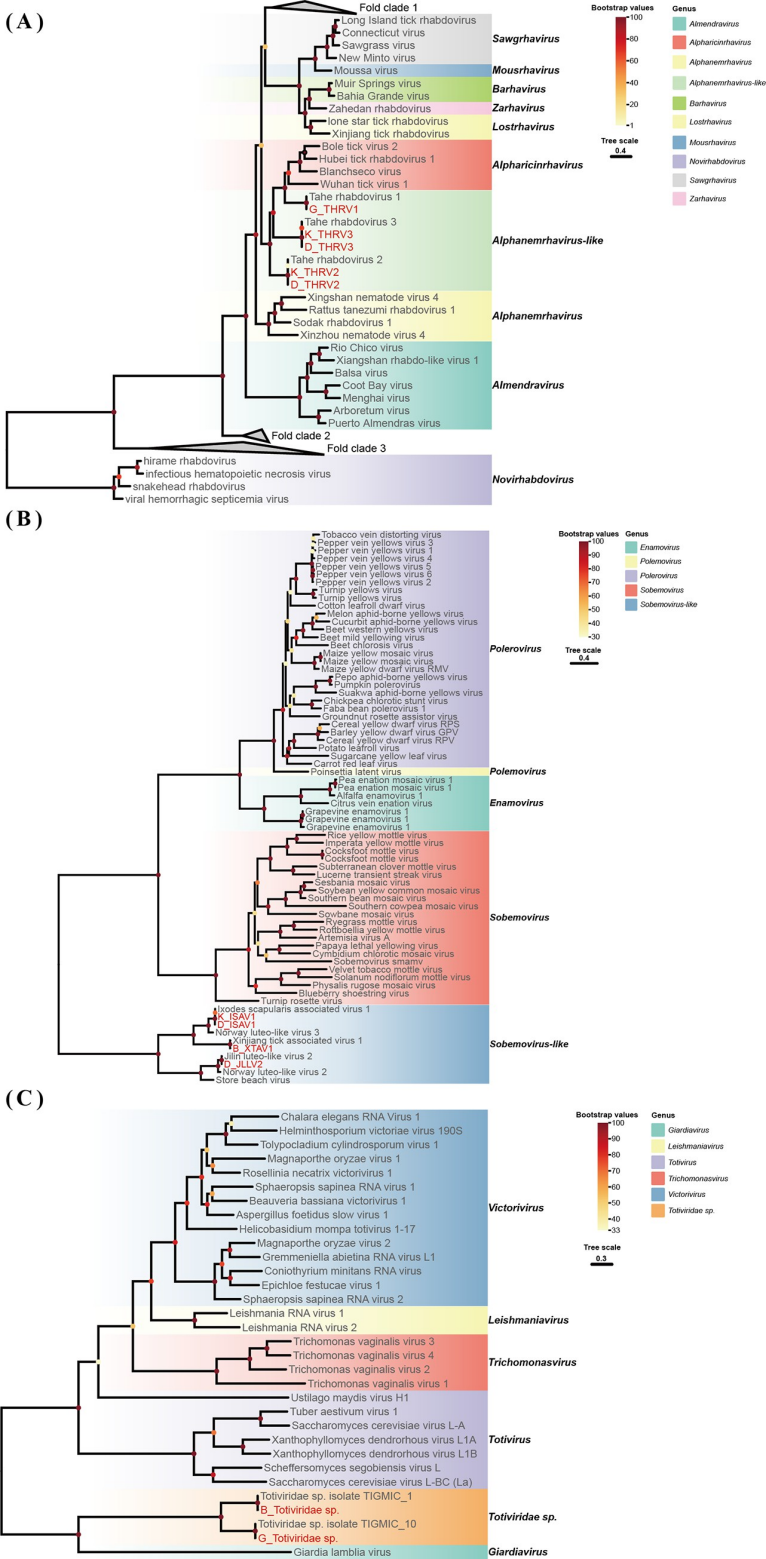
Phylogenetic analysis of the novel strains of *Rhabdoviridae*, *Solemoviridae* and *Totiviridae*. ML tree of representative viruses based on the RdRp genes. (A) ML tree of *Rhabdoviridae* (**Fold clade 1** includes *Hapavirus*, *Arurhavirus*, *Curiovirus*, *Ephemerovirus*, *Tibrovirus*, *Vesiculovirus*, *Sprivivirus*, *Perhabdovirus*, *Siniperhavirus*, *Cetarhavirus*, *Scophrhavirus*, *Sigmaviris*, *Alphapaprhavirus*, *Ohlsrhavirus*, *Merhavirus*, *Caligrhavirus*, *Sunrhavirus*, *Sripuvirus*, *Tupavirus*, *Ledantevirus*, and *Thriprhavirus*. **Fold clade 2** includes *Lyssavirus*, *Replylivirus*, and *Amplylivirus*. **Fold clade 3** includes *Betanucleorhabdovirus*, *Dichorhavirus*, *Alphanucleorhabdovirus*, *Gammanucleorhabdovirus*, *Varicosavirus*, *Cytorhabdovirus*, *Alphahymrhavirus*, *Betanemrhavirus*, *Alphacrustrhavirus*, *Betaricinrhavirus*, *Aphadrosrhavirus*, *Betahymrhavirus*, and *Betanemrhavirus*). (B) ML tree of *Solemoviridae*. (C) ML tree of *Totiviridae*. The viruses newly identified in this study and previously identified viruses are shown in red and black, respectively. The best-fit amino acid substitution model for each phylogenetic tree was LG + F + R10 for *Rhabdoviridae*, LG + F + R5 for *Solemoviridae*, and LG + F + I + G4 for *Totiviridae*.

In the phylogenetic tree constructed on the basis of the amino acid sequences of the viral RdRp proteins, the sequences representative of this study were clustered with the corresponding published viral sequences obtained from the NCBI database (Figs 2–4).

### Phylogenetic analysis of unclassified *Bunyavirales* sequences

The order *Bunyavirales* encompasses a diverse array of viruses, many of which are arthropod-borne and capable of causing significant disease in humans and animals, including the Crimean-Congo haemorrhagic fever virus (CCHFV), Hantavirus, and Rift Valley fever virus (RVFV) [34–36]. In this study, a contig belonging to Volzhskoe tick virus, an unclassified member of the *Bunyavirales*, was identified in *H*. *marginatum* ticks; the strain from which this contig was derived showed 87.3% nucleotide similarity to a previously identified strain of *H*. *marginatum* from Astrakhan, Russia, and it clustered in the same branch of the phylogenetic tree (Table 2 and Fig 2A**)**.

### Phylogenetic analysis of *Flaviviridae* sequences

The family *Flaviviridae* is extensive and includes numerous significant human pathogens [37,38]. Three viruses belonging to the *Flaviviridae* were identified in three tick species. These viruses include ALSV and TBEV, both of which are associated with human disease; they were found in *I*. *persulcatus* ticks from Hulun Buir and Hinggan League. The nucleotide identities of these viruses with other strains of *I*. *persulcatus* ranged from 98.0% to 99.6% for TBEV and from 95.8% to 99.5% for ALSV. Additionally, a *Pestivirus-like* virus, Bole tick virus 4, was detected in *D*. *nuttalli* and *H*. *marginatum*; it had nucleotide identities of 93.2%–97.5% with other Bole tick virus 4 strains. These sequences all clustered with published sequences (Table 2 and Fig 2B**)**.

### Phylogenetic analysis of *Nairoviridae* sequences

Members of the family *Nairoviridae* consist of three negative-sense, single-stranded RNA segments [39]. *Orthonairovirus* is a genus within the *Nairoviridae*. These viruses are found in arthropods and are transmitted to mammals by ticks [40]. BJNV and YZV were discovered in *I*. *persulcatus* ticks through NGS. BJNV sharing nucleotide identities of 98.4%–99.8% with other strains was detected in *I*. *persulcatus* from both Hulun Buir and Hinggan League. YZV was detected only in *I*. *persulcatus* ticks from Hulun Buir; it had nucleotide identities of 98.6%–99.0% with other strains (Table 2 and Fig 2C**)**.

### Phylogenetic analysis of *Chuviridae* sequences

The genus *Mivirus* belongs to the newly classified family *Chuviridae*, a recently recognized viral family within the order Jingchuvirales [41,42]. Two *Mivirus* viruses and one *Nigecruvirus* virus were identified in ticks by NGS. NUMV showing 99.8%–99.9% nucleotide identity with other strains was detected in *I*. *persulcatus* ticks from Hulun Buir and Hinggan League. Bole tick virus 3 was derived from only *H*. *marginatum* ticks; it showed 98.6% nucleotide similarity to strains isolated from *Hyalomma asiaticum*. Taiga tick nigecruvirus was obtained from *I*. *persulcatus* ticks from Hinggan League and presented 98.0% nucleotide identity with other strains (Table 2 and Fig 3A**)**.

### Phylogenetic analysis of *Partitiviridae* sequences

A *Deltapartitivirus-like* virus, Jilin partita-like virus 1, belonging to the family *Partitiviridae* was harvested from *I*. *persulcatus* ticks from Hinggan League and showed nucleotide identities of 99.7% with other strains (Table 2 and Fig 3B**)**. The family *Partitiviridae* is known for its double-stranded RNA genomes and typically infects plants and fungi [43].

### Phylogenetic analysis of *Phenuiviridae* sequences

The genus *Phlebovirus* belongs to the family *Phenuiviridae*; it infects mammals, including livestock and humans, and is transmitted by infected arthropods [44,45]. Three viruses belonging to the *Phenuiviridae* were found in *I*. *persulcatus* ticks: one *Phlebovirus*, MKWV, and two *Ixoviruses*, Onega tick phlebovirus and Sara tick phlebovirus. The nucleotide identities of these strains were 89.1%–98.8%, 99.6%–100.0%, and 99.6%–100.0%, respectively (Table 2 and Fig 3C).

### Phylogenetic analysis of *Rhabdoviridae* sequences

The family *Rhabdoviridae*, a member of the order *Mononegavirales*, includes important pathogens that affect humans, livestock, fish, and agricultural crops [46]. Rhabdoviruses are found in invertebrates such as arthropods, some of which serve as hosts or vectors for transmission to other animals or plants [47]. We identified several viral contigs belonging to *Rhabdoviridae*; all were *Alphanemrhavirus-like* viruses. The strains included Tahe rhabdovirus 1 from *H*. *concinna* ticks, and Tahe rhabdovirus 2 and Tahe rhabdovirus 3 from *I*. *persulcatus* ticks from Hulun Buir and Hinggan League; the nucleotide identities of these viruses with other strains were 99.6%, 98.7%–99.2%, and 99.7–99.9%, respectively (Table 2 and Fig 4A).

### Phylogenetic analysis of *Solemoviridae* sequences

*Solemoviridae* is a recently recognized family of plant-infecting viruses. *Solemoviridae* are transmitted abiotically via mechanical damage, via asexual reproduction or through soil or insects [48]. In this study, we identified several viral contigs belonging to the *Solemoviridae*. Xinjiang tick-associated virus 1 was identified in *D*. *nuttalli* ticks, and Ixodes scapularis-associated virus 1 and Jilin luteo-like virus 2 were discovered in *I*. *persulcatus* ticks. The nucleotide identities of these viruses with other strains were 98.9%, 98.5%–99.2%, and 98.9%, respectively (Table 2 and Fig 4B).

### Phylogenetic analysis of *Totiviridae* sequences

The *Totiviridae* are double-stranded RNA viruses that commonly infect fungi and protozoa [49]. In this study, Totiviridae sp. showing 96.9%–98.6% nucleotide identity with previously discovered strains were detected in *H*. *concinna* (Table 2 and Fig 4C).

### Statistical analysis of the virus detection results

In this study, important pathogenic viruses were identified in RNA libraries by NGS; each sample of collected ticks was further subjected to PCR combined with sequencing to identify the RNA viruses it carried and to analyse the distribution and prevalence of the viruses. The primers used for amplification are listed in S3 Table. The nucleotide sequences of the identified RNA viruses amplified via PCR have been submitted to NCBI (S4 Table).

A total of 20 viruses representing more than eight different families were confirmed to be present in the ticks collected in this study. RNA viruses were detected in 76 (58.5%) of the 130 tick pools that underwent molecular screening (Table 3). Thirteen viruses were detected in *I*. *persulcatus* collected in Hinggan League. Among these tick samples, Jilin partiti-like virus 1 had the lowest prevalence at 0.5% (3/26), whereas the emerging zoonotic pathogen BJNV had the highest prevalence at 7.7% (23/26). Other tick-borne viruses with potential relevance to human and livestock diseases included TBEV, ALSV, NUMV, and MKWV; the collected ticks were infected with these viruses at rates of 1.1% (6/26), 1.4% (7/26), 4.7% (17/26), and 1.7% (8/26), respectively.

**Table 3:** PCR survey results for the viral testing of ticks collected in Inner Mongolia, China, from 2021 to 2023. The rates at which ticks were infected with viruses were calculated using the bias-corrected MLE method in PooledInfRate software, version 4.0; 95% confidence intervals (CIs) are presented in brackets.

Virus species	Total no. (%) ticks positive [95% CI]					
	Hinggan League	Bayan Nur		Hulun Buir		
	*Ixodes persulcatus*	*Dermacentor nuttalli*	*Hyalomma marginatum*	*Haemaphysalis concinna*	*Ixodes persulcatus*	
Mukawa virus	8/26 (1.67) [0.78–3.29]	0	0	0	1/14 (0.39) [0.02–1.92]	
Alongshan virus	7/26 (1.40) [0.62–2.86]	0	0	0	3/14 (1.26) [0.34–3.46]	
Nuomin virus	17/26 (4.68) [2.86–7.65]	0	0	0	2/14 (0.79) [0.15–2.57]	
Beiji nairovirus	23/26 (7.70) [5.20–12.02]	0	0	0	12/14 (8.73) [4.93–16.73]	
Sara tick phlebovirus	20/26 (6.14) [3.95–9.78]	0	0	0	6/14 (2.77) [1.18–5.80]	
Tahe rhabdovirus 3	13/26 (2.86) [1.65–4.80]	0	0	0	1/14 (0.38) [0.02–1.84]	
Bole tick virus 4	0	6/30 (5.20) [2.24–10.37]	20/50 (17.4) [11.34–25.49]	0		0
Tick-borne encephalitis virus	6/26 (1.11) [0.46–2.33]	0	0	0		0
Onega tick phlebovirus	15/26 (3.47) [2.11–5.62]	0	0	0		3/14 (1.21) [0.33–3.26]
Tahe rhabdovirus 2	5/26 (0.95) [0.35–2.16]	0	0	0		0
Jilin luteo-like virus 2	10/26 (2.22) [1.13–4.10]	0	0	0		0
Tahe rhabdovirus 1	0	0	0	1/10 (4.83) [0.29–21.90]		0
Ixodes scapularis associated virus 1	5/26 (0.90) [0.34–2.02]	0	0	0		0
Yezo virus	0	0	0	0		1/14 (0.38) [0.02–1.89]
Jilin partiti-like virus 1	3/26 (0.50) [0.14–1.34]	0	0	0		0
Xinjiang tick associated virus 1	0	1/30 (0.82) [0.05–3.92]	0	0		0
Totiviridae sp.	0	1/30 (0.82) [0.05–3.92]	0	0		0
Bole tick virus 3	0	0	2/50 (1.36) [0.25–4.39]	0		0
Taiga tick nigecruvirus	5/26 (0.89) [0.34–1.98]	0	0	0		0
Volzhskoe tick virus	0	0	14/50 (10.10) [6.07–15.67]	0		0

Eight viruses, including five potentially pathogenic viruses, were detected in *I*. *persulcatus* from Hulun Buir. YZV had the lowest prevalence at 0.38% (1/14), followed by NUMV at 0.8% (2/14), ALSV at 1.3% (3/14), Sara tick phlebovirus at 0.4% (1/14), and BJNV, which had the highest prevalence at 8.7% (12/14).

Specimens of *D*. *nuttalli* and *H*. *marginatum* collected from Bayan Nur were found to carry three different viruses each. Both of these species were coinfected with Bole tick virus 4 at rates of 5.2% (6/30) and 17.4% (20/50), respectively. Volzhskoe tick virus and Bole tick virus 3 were detected in *H*. *marginatum* in China for the first time, with infection rates of 10.1% (14/50) and 1.4% (2/50), respectively. Xinjiang tick-associated virus 1 and Totiviridae sp. were detected only in *D*. *nuttalli*; the infection rate for each of these viruses was 0.8% (1/30).

A statistical comparison of the carriage rates of concurrently detected viruses in *I*. *persulcatus* from two different regions revealed significant disparities (Table 4). The prevalence of ALSV remained consistent across regions. However, substantial regional discrepancies were observed for Sara tick phlebovirus and Onega tick phlebovirus, both of which belong to the *Phenuiviridae*; these viruses exhibited notably greater prevalence in ticks collected from Hinggan League than in ticks collected from Hulun Buir. Additionally, BJNV, an *Orthonairovirus* from the *Nairoviridae* family, presented the highest prevalence among the cases of concurrent infection; it displayed a homogeneous distribution across the studied tick populations, indicating widespread prevalence. Conversely, significant disparities in the distributions of NUMV and Tahe rhabdovirus 3, which belong to the *Chuviridae* and *Rhabdoviridae*, respectively, were observed; these viruses were more prevalent in Hinggan League than in Hulun Buir.

**Table 4:** Prevalence of viruses detected in *I*. *persulcatus* collected in two cities.

Viruses	Pool positivity rates of viruses in *I*. *persulcatus*		X^2^-value	P
	Hinggan League	Hulun Buir		
Mukawa virus	8/26	1/14	2.913	0.088
Sara tick phlebovirus	20/26	6/14	10.128	0.001
Onega tick phlebovirus	15/26	3/14	4.835	0.028
Alongshan virus	7/26	3/14	0.147	0.702
Beiji nairovirus	23/26	12/14	0.063	0.802
Tahe rhabdovirus 3	13/26	1/14	5.584	0.018
Nuomin virus	17/26	2/14	9.528	0.002

## Discussion

In this study, we delineated the diverse constellation of RNA viromes harboured by several tick species in Inner Mongolia, China. By utilizing high-throughput transcriptome sequencing, we identified a wide range of RNA viruses in *I*. *persulcatus*, *D*. *nuttalli*, *H*. *marginatum*, and *H*. *concinna*. The 20 distinct viral species represented at least 8 virus families, including *Chuviridae*, *Flaviviridae*, *Solemoviridae*, *Nairoviridae*, *Partitiviridae*, *Phenuiviridae*, *Rhabdoviridae*, and *Totiviridae*.

PCR validation reinforced the data obtained through NGS, unequivocally confirming the presence of a diverse array of viruses within the tick populations subjected to analysis. Among the detected viruses, NUMV [20], YZV [50], TBEV [51], ALSV [52], and BJNV [53] have been recognized as pathogens that are capable of infecting humans. These findings not only reaffirm the findings of previous studies of viromes in Northeast China [15,20,54] but also emphasize the widespread presence of RNA viruses in tick populations. Notably, ALSV has been detected in tick-bitten patients and in *I*. *persulcatus* in Inner Mongolia and Heilongjiang [52]. Additionally, viral RNA and virus-specific antibodies have been identified in sheep and cattle in Hulunbuir, northeastern Inner Mongolia [55]. The detection of ALSV in *I*. *persulcatus* ticks collected from Hinggan League and Hulun Buir, Inner Mongolia, further confirms previous findings. Tick-borne encephalitis (TBE) has been shown to be endemic in China, including the three mountainous areas of Heilongjiang, Jilin, Inner Mongolia, and Xinjiang [24,56]. Inner Mongolia, especially the forested and grassland environments of that region, plays a particularly important role in the transmission of tick-borne encephalitis virus (TBEV). These areas provide conditions that favour the survival and proliferation of ticks, facilitating the spread of TBEV. In this study, TBEV was detected in *I*. *persulcatus* ticks collected from the Hinggan League of Inner Mongolia. Liu et al. previously detected TBEV in northeastern China via metagenomic techniques [15]. However, the results of the current study suggest that the natural foci of TBEV in these regions may be more widespread than was previously believed. The current findings underscore the importance of ongoing surveillance for tick-borne viruses and the need for public health interventions. Phleboviruses infect mammals, including livestock and humans, and are transmitted by infected mosquitoes, phlebotomine sandflies, and ticks [57]. Studies have shown that Mukawa virus can replicate in mammalian cells, indicating that there is potential for its zoonotic transmission [58]. In this study, MKWV was detected in *I*. *persulcatus* ticks collected from two locations. Previous studies have detected these viruses in ticks of various classes in various regions, but the current study provides a more comprehensive understanding of the distribution of these viruses as well as of their prevalence in different tick species. Notably, the Volzhskoe tick virus, previously detected only in *I*. *persulcatus* ticks from northern China [20], is here reported for the first time to be present in *H*. *marginatum* ticks in China. Although the pathogenicity of this virus remains unknown, the findings suggest that the virus may have a broader geographical distribution than was previously suspected and that various tick species may act as vectors of the virus within China.

The utilization of PCR-based methods to detect viruses in tick populations has revealed significant differences in viral prevalence among tick species and in different geographical locations. In Hinggan League, *I*. *persulcatus* tick pools presented an exceptionally high prevalence of viral infection; 100% (26/26) of the collected tick pools tested positive for viruses. In Hulun Buir, the virus positivity rate in *I*. *persulcatus* tick pools was also high, reaching 92.9% (13/14). In contrast, in Bayan Nur, the virus infection rates in the *D*. *nuttalli* and *H*. *marginatum* tick pools were lower, 20.0% (6/30) and 60.0% (30/50), respectively. Notably, in Hulun Buir, only one *H*. *concinna* sample pool tested positive for viruses, yielding a virus detection rate of 10.0% (1/10). It is noteworthy that the elevated number of viruses detected in *I*. *persulcatus* may be attributed to the substantially larger sample size collected. Furthermore, *I*. *persulcatus* were gathered as free-living ticks from forest and shrub environments, in contrast to other species, which were collected directly from host animals. These findings reflect differences in the capacities of various tick species to serve as virus vectors. Consistent with previous studies, *I*. *persulcatus* ticks were found to carry the most diverse range of viruses [59].

The species-specific distribution of tick-borne viruses is further exemplified by the exclusive presence of certain viruses in particular tick hosts. Xinjiang tick-associated virus 1 and Totiviridae sp. were detected only in *D*. *nuttalli* ticks. Bole tick virus 3 and Volzhskoe tick viruses were uniquely identified in *H*. *marginatum* ticks, whereas Tahe rhabdovirus 1 was isolated exclusively from *H*. *concinna*. In contrast, *I*. *persulcatus* ticks were identified as hosts of 14 other distinct viruses, including 5 tick-borne viruses known to cause illness in humans as well as other potentially pathogenic viruses.

A statistical analysis of the prevalence of specific viruses in *I*. *persulcatus* ticks from two distinct regions revealed comparable rates of infection by ALSV and BJNV in the two regions. However, a marked discrepancy was observed in the prevalence of Sara tick phlebovirus, Onega tick phlebovirus, NUMV, and Tahe rhabdovirus 3, with ticks from Hinggan League demonstrating a significantly greater rate of infection with these viruses than ticks from Hulun Buir. The survival and reproduction of ticks, which are important vector organisms, are directly influenced by environmental conditions. Consequently, the distributions of tick species and the viruses they carry vary across different ecosystems. The Hinggan League area has a high density of forests that are predominantly coniferous and provide a stable habitat for ticks. The diverse forest vegetation and the humid environment of this region favour tick survival and reproduction. Additionally, the deciduous and humus layers of vegetation in the forest provide suitable habitats for ticks. In contrast, the Hulun Buir area is primarily a steppe ecosystem in which there is relatively sparse vegetation cover, especially during the dry season when the vegetation density is lowest. The open environment and greater temperature fluctuations in the grassland environment are less conducive to tick survival and reproduction than are conditions in the forest environment. Owing to their stable and diverse ecosystems, Hinggan League and Hulun Buir offer favourable habitat conditions for a diverse range of tick species and the viruses they carry. Conversely, the arid environment of Bayan Nur restricts the diversity of tick species and their viruses. The findings suggest that the ecological diversity of Inner Mongolia significantly affects the distribution of RNA viruses in ticks. In addition, changes in natural environmental conditions alter tick habitats and thereby impact the ecology of tick-borne viruses [60]. The ecological data gathered in this study will aid future research on virus transmission through advanced methods such as mathematical modelling and machine learning [61,62]. Notably, the prominent presence of BJNV, which emerged as the pathogen with the highest prevalence in ticks in Inner Mongolia, indicated a widespread distribution of this virus within the *I*. *persulcatus* populations of both regions. Since ticks cannot travel long distances, they are likely to acquire the viruses they harbour from local organisms with which they interact. This highlights both the potential for BJNV to pose a significant public health concern and the importance of ongoing surveillance for tick-borne pathogens.

In conclusion, this study utilized NGS to investigate the spectrum of viruses present in tick populations across various regions of Inner Mongolia. Validation of the NGS results was performed via PCR combined with Sanger sequencing; in this way, the prevalence of these viruses in various tick species was determined. The detection of these viruses not only underscores the diversity of RNA viruses carried by ticks in Inner Mongolia but also reveals the variations in their distributions in different regions and different tick hosts. However, it is important to acknowledge the limitations of this study. Some other causative viruses previously reported in Inner Mongolia, such as the severe fever with thrombocytopenia syndrome virus (SFTSV) [54] and SGLV [15], were not detected in this investigation. Our investigation may not encompass all tick species and geographical areas in Inner Mongolia and may have potentially overlooked certain viral hosts. Moreover, our understanding of the epidemiology of these viruses and their adaptability to hosts remains superficial. Future research should aim to broaden the sample range by including a greater diversity of tick species and geographical locations and by conducting more in-depth studies into the ecology and evolution of viruses carried by ticks.

## Conclusions

The findings of this study revealed significant diversity among RNA viruses carried by ticks collected from various geographical regions in Inner Mongolia, China. To detect alterations in pathogen distribution effectively, ongoing surveillance is urgently needed. The changing natural environment is altering tick habitats, thereby affecting the distribution of the viruses they transmit. Our findings across different ecosystems and climates lay a foundation for research on predicting the spatiotemporal distribution and transmission of viruses.

## Supporting information

S1 Table Grouping and pooling information on the studied adult ticks. (DOC)

S2 Table Viruses identified from each library in the present study. (DOC)

S3 Table Primer sequences used in virus detection. (DOC)

S4 Table Accession numbers of viral sequences amplified by PCR. (DOC)

S1 File Data used in phylogenetic tree construction for each family, including aligned sequences, software logs, best-fit substitution models, and phylogenetic trees. (ZIP)
