# Supplementary material for: Metatranscriptomic analysis reveals the diversity of RNA viruses in ticks in Inner Mongolia, China
Source: PLoS Negl Trop Dis. 2024 Dec 11;18(12):e0012706. doi: 10.1371/journal.pntd.0012706 (PMC11634002; doi:10.1371/journal.pntd.0012706)
Supplement: S1 Table — (DOC) [file pntd.0012706.s001.doc]

**S1 Table. Grouping and pooling information on the studied adult ticks.**

| Pools name | Life stages | Collection sites | Host species | Ticks number |
| --- | --- | --- | --- | --- |
| B1 | adults | Bayan Nur | Dermacentor nuttalli | 1 |
| B2 | adults | Bayan Nur | Dermacentor nuttalli | 1 |
| B3 | adults | Bayan Nur | Dermacentor nuttalli | 1 |
| B4 | adults | Bayan Nur | Dermacentor nuttalli | 4 |
| B5 | adults | Bayan Nur | Dermacentor nuttalli | 4 |
| B6 | adults | Bayan Nur | Dermacentor nuttalli | 4 |
| B7 | adults | Bayan Nur | Dermacentor nuttalli | 6 |
| B8 | adults | Bayan Nur | Dermacentor nuttalli | 6 |
| B9 | adults | Bayan Nur | Dermacentor nuttalli | 3 |
| B10 | adults | Bayan Nur | Dermacentor nuttalli | 1 |
| B11 | adults | Bayan Nur | Dermacentor nuttalli | 1 |
| B12 | adults | Bayan Nur | Dermacentor nuttalli | 6 |
| B13 | adults | Bayan Nur | Dermacentor nuttalli | 1 |
| B14 | adults | Bayan Nur | Dermacentor nuttalli | 6 |
| B15 | adults | Bayan Nur | Dermacentor nuttalli | 8 |
| B16 | adults | Bayan Nur | Dermacentor nuttalli | 8 |
| B17 | adults | Bayan Nur | Dermacentor nuttalli | 1 |
| B18 | adults | Bayan Nur | Dermacentor nuttalli | 4 |
| B19 | adults | Bayan Nur | Dermacentor nuttalli | 6 |
| B20 | adults | Bayan Nur | Dermacentor nuttalli | 1 |
| B21 | adults | Bayan Nur | Dermacentor nuttalli | 6 |
| B22 | adults | Bayan Nur | Dermacentor nuttalli | 9 |
| B23 | adults | Bayan Nur | Dermacentor nuttalli | 13 |
| B24 | adults | Bayan Nur | Dermacentor nuttalli | 11 |
| B25 | adults | Bayan Nur | Dermacentor nuttalli | 2 |
| B26 | adults | Bayan Nur | Dermacentor nuttalli | 1 |
| B27 | adults | Bayan Nur | Dermacentor nuttalli | 1 |
| B28 | adults | Bayan Nur | Dermacentor nuttalli | 2 |
| B29 | adults | Bayan Nur | Dermacentor nuttalli | 3 |
| B30 | adults | Bayan Nur | Dermacentor nuttalli | 1 |
| D1 | adults | Hinggan League | Ixodes persulcatus | 31 |
| D2 | adults | Hinggan League | Ixodes persulcatus | 71 |
| D3 | adults | Hinggan League | Ixodes persulcatus | 52 |
| D4 | adults | Hinggan League | Ixodes persulcatus | 47 |
| D6 | adults | Hinggan League | Ixodes persulcatus | 20 |
| D7 | adults | Hinggan League | Ixodes persulcatus | 10 |
| D8 | adults | Hinggan League | Ixodes persulcatus | 15 |
| D9 | adults | Hinggan League | Ixodes persulcatus | 15 |
| D10 | adults | Hinggan League | Ixodes persulcatus | 15 |
| D11 | adults | Hinggan League | Ixodes persulcatus | 20 |
| D12 | adults | Hinggan League | Ixodes persulcatus | 19 |
| D13 | adults | Hinggan League | Ixodes persulcatus | 16 |
| D14 | adults | Hinggan League | Ixodes persulcatus | 17 |
| D15 | adults | Hinggan League | Ixodes persulcatus | 17 |
| D16 | adults | Hinggan League | Ixodes persulcatus | 17 |
| D17 | adults | Hinggan League | Ixodes persulcatus | 20 |
| D18 | adults | Hinggan League | Ixodes persulcatus | 21 |
| D19 | adults | Hinggan League | Ixodes persulcatus | 15 |
| D20 | adults | Hinggan League | Ixodes persulcatus | 15 |
| D21 | adults | Hinggan League | Ixodes persulcatus | 17 |
| D22 | adults | Hinggan League | Ixodes persulcatus | 15 |
| D23 | adults | Hinggan League | Ixodes persulcatus | 15 |
| D24 | adults | Hinggan League | Ixodes persulcatus | 20 |
| D25 | adults | Hinggan League | Ixodes persulcatus | 17 |
| D26 | adults | Hinggan League | Ixodes persulcatus | 20 |
| D27 | adults | Hinggan League | Ixodes persulcatus | 60 |
| E1 | adults | Bayan Nur | Hyalomma marginatum | 2 |
| E2 | adults | Bayan Nur | Hyalomma marginatum | 2 |
| E3 | adults | Bayan Nur | Hyalomma marginatum | 1 |
| E4 | adults | Bayan Nur | Hyalomma marginatum | 6 |
| E5 | adults | Bayan Nur | Hyalomma marginatum | 3 |
| E6 | adults | Bayan Nur | Hyalomma marginatum | 5 |
| E7 | adults | Bayan Nur | Hyalomma marginatum | 1 |
| E8 | adults | Bayan Nur | Hyalomma marginatum | 1 |
| E9 | adults | Bayan Nur | Hyalomma marginatum | 3 |
| E10 | adults | Bayan Nur | Hyalomma marginatum | 1 |
| E11 | adults | Bayan Nur | Hyalomma marginatum | 5 |
| E12 | adults | Bayan Nur | Hyalomma marginatum | 2 |
| E13 | adults | Bayan Nur | Hyalomma marginatum | 1 |
| E14 | adults | Bayan Nur | Hyalomma marginatum | 4 |
| E15 | adults | Bayan Nur | Hyalomma marginatum | 3 |
| E16 | adults | Bayan Nur | Hyalomma marginatum | 6 |
| E17 | adults | Bayan Nur | Hyalomma marginatum | 7 |
| E18 | adults | Bayan Nur | Hyalomma marginatum | 1 |
| E19 | adults | Bayan Nur | Hyalomma marginatum | 6 |
| E20 | adults | Bayan Nur | Hyalomma marginatum | 8 |
| E21 | adults | Bayan Nur | Hyalomma marginatum | 6 |
| E22 | adults | Bayan Nur | Hyalomma marginatum | 1 |
| E23 | adults | Bayan Nur | Hyalomma marginatum | 1 |
| E24 | adults | Bayan Nur | Hyalomma marginatum | 1 |
| E25 | adults | Bayan Nur | Hyalomma marginatum | 4 |
| E26 | adults | Bayan Nur | Hyalomma marginatum | 11 |
| E27 | adults | Bayan Nur | Hyalomma marginatum | 3 |
| E28 | adults | Bayan Nur | Hyalomma marginatum | 1 |
| E29 | adults | Bayan Nur | Hyalomma marginatum | 1 |
| E30 | adults | Bayan Nur | Hyalomma marginatum | 4 |
| E31 | adults | Bayan Nur | Hyalomma marginatum | 1 |
| E32 | adults | Bayan Nur | Hyalomma marginatum | 3 |
| E33 | adults | Bayan Nur | Hyalomma marginatum | 1 |
| E34 | adults | Bayan Nur | Hyalomma marginatum | 3 |
| E35 | adults | Bayan Nur | Hyalomma marginatum | 2 |
| E36 | adults | Bayan Nur | Hyalomma marginatum | 6 |
| E37 | adults | Bayan Nur | Hyalomma marginatum | 4 |
| E38 | adults | Bayan Nur | Hyalomma marginatum | 4 |
| E39 | adults | Bayan Nur | Hyalomma marginatum | 4 |
| E40 | adults | Bayan Nur | Hyalomma marginatum | 1 |
| E41 | adults | Bayan Nur | Hyalomma marginatum | 2 |
| E42 | adults | Bayan Nur | Hyalomma marginatum | 4 |
| E43 | adults | Bayan Nur | Hyalomma marginatum | 2 |
| E44 | adults | Bayan Nur | Hyalomma marginatum | 1 |
| E45 | adults | Bayan Nur | Hyalomma marginatum | 1 |
| E46 | adults | Bayan Nur | Hyalomma marginatum | 1 |
| E47 | adults | Bayan Nur | Hyalomma marginatum | 1 |
| E48 | adults | Bayan Nur | Hyalomma marginatum | 3 |
| E49 | adults | Bayan Nur | Hyalomma marginatum | 1 |
| E50 | adults | Bayan Nur | Hyalomma marginatum | 2 |
| G1 | adults | Hulun Buir | Haemaphysalis concinna | 4 |
| G2 | adults | Hulun Buir | Haemaphysalis concinna | 5 |
| G3 | adults | Hulun Buir | Haemaphysalis concinna | 5 |
| G4 | adults | Hulun Buir | Haemaphysalis concinna | 1 |
| G5 | adults | Hulun Buir | Haemaphysalis concinna | 1 |
| G6 | adults | Hulun Buir | Haemaphysalis concinna | 1 |
| G7 | adults | Hulun Buir | Haemaphysalis concinna | 1 |
| G8 | adults | Hulun Buir | Haemaphysalis concinna | 1 |
| G9 | adults | Hulun Buir | Haemaphysalis concinna | 1 |
| G10 | adults | Hulun Buir | Haemaphysalis concinna | 1 |
| K1 | adults | Hulun Buir | Ixodes persulcatus | 15 |
| K2 | adults | Hulun Buir | Ixodes persulcatus | 14 |
| K3 | adults | Hulun Buir | Ixodes persulcatus | 31 |
| K4 | adults | Hulun Buir | Ixodes persulcatus | 28 |
| K5 | adults | Hulun Buir | Ixodes persulcatus | 20 |
| K6 | adults | Hulun Buir | Ixodes persulcatus | 15 |
| K7 | adults | Hulun Buir | Ixodes persulcatus | 15 |
| K8 | adults | Hulun Buir | Ixodes persulcatus | 18 |
| K9 | adults | Hulun Buir | Ixodes persulcatus | 20 |
| K10 | adults | Hulun Buir | Ixodes persulcatus | 12 |
| K11 | adults | Hulun Buir | Ixodes persulcatus | 14 |
| K12 | adults | Hulun Buir | Ixodes persulcatus | 13 |
| K13 | adults | Hulun Buir | Ixodes persulcatus | 26 |
| K14 | adults | Hulun Buir | Ixodes persulcatus | 19 |
