# Supplementary material for: Metatranscriptomic analysis reveals the diversity of RNA viruses in ticks in Inner Mongolia, China
Source: PLoS Negl Trop Dis. 2024 Dec 11;18(12):e0012706. doi: 10.1371/journal.pntd.0012706 (PMC11634002; doi:10.1371/journal.pntd.0012706)
Supplement: S2 Table — (DOC) [file pntd.0012706.s002.doc]

**S2 Table. Viruses identified from each library in the present study.**

| **Family** | **Genus** | | **Virus (abbreviation)** | **Length（nt）** | **Relative abundance (log10(RPM) )** | **Closest relative (accession no)** | **Query Cover** | **Per.Ident** |
| --- | --- | --- | --- | --- | --- | --- | --- | --- |
| Bunyavirales | unclassified Bunyavirales | E_VSTV | | 1328 | 1.68 | Volzhskoe tick virus strain Hun/Hy_marginatum/08-2021 (OQ849227.1) | 99.0% | 87.3% |
| Chuviridae | Mivirus | D_NUMV | | 8780 | 3.13 | Nuomin virus strain SL4 (ON408179.1) | 99.0% | 99.8% |
| Chuviridae | Nigecruvirus | D_TGTNV | | 11447 | 1.96 | Taiga tick nigecruvirus isolate HLJ-IP-18 (OP863301.1) | 99.0% | 98.0% |
| Chuviridae | Mivirus | E_BLTV3 | | 1293 | 0.22 | Bole Tick Virus 3 isolate GSC346MV (MZ244265.1) | 100.0% | 98.6% |
| Chuviridae | Mivirus | K_NUMV | | 11041 | 2.16 | Nuomin virus strain SL4 ( ON408179.1) | 99.0% | 99.8% |
| Flaviviridae | Pestivirus-like | B_BLTV4 | | 16653 | 4.92 | Flaviviridae sp.isolate TlGMlC_1 (ON811700.1) | 99.0% | 97.5% |
| Flaviviridae | Flavivirus | D_TBEV | | 11083 | 2.68 | Tick-borne encephalitis virus strain TSA-18 (MN114635.1) | 100.0% | 98.0% |
| Flaviviridae | Pestivirus-like | E_BLTV4 | | 7389 | 4.73 | Bole tick virus 4 strain Iftin/H.dromedarii/2018 (MW561976.1) | 98.0% | 93.2% |
| Flaviviridae | Jingmenvirus | K_ALSV | | 2277 | 2.39 | Alongshan virus strain NE-TH4 segment 1 (ON408067.1) | 100.0% | 98.3% |
| Flaviviridae | Jingmenvirus | K_ALSV | | 1446 | 3.01 | Alongshan virus strain H3 (MH158416.1) | 100.0% | 99.2% |
| Flaviviridae | Jingmenvirus | K_ALSV | | 2310 | 2.82 | Alongshan virus strain NE-TH4 segment 3 (ON408069.1) | 100.0% | 98.8% |
| Flaviviridae | Jingmenvirus | K_ALSV | | 2345 | 2.63 | Alongshan virus strain H3 (MH158418.1) | 100.0% | 99.5% |
| Flaviviridae | Flavivirus | K_TBEV | | 11037 | 2.10 | Tick-borne encephalitis virus strain HLB-T74 (MN615727.1) | 99.0% | 99.6% |
| Nairoviridae | Norwavirus | D_BJNV | | 11862 | 4.54 | Beiji nariovirus strain NE-TH3 segment L (ON408098.1) | 99.0% | 99.8% |
| Nairoviridae | Norwavirus | D_BJNV | | 3480 | 3.27 | Beiji nariovirus strain NE-YC3 segment S (ON408105.1) | 88.0% | 98.4% |
| Nairoviridae | Norwavirus | K_BJNV | | 13243 | 3.18 | Beiji nariovirus strain NE-TH3 segment L (ON408098.1) | 100.0% | 99.4% |
| Nairoviridae | Norwavirus | K_BJNV | | 1941 | 2.54 | Beiji nariovirus strain NE-TH4 segment S (ON408101.1) | 100.0% | 99.0% |
| Nairoviridae | Orthonairovirus | K_YZV | | 12118 | 1.52 | Yezo virus isolate TIGMIC_3 (ON811933.1) | 99.0% | 98.7% |
| Nairoviridae | Orthonairovirus | K_YZV | | 3040 | 0.48 | Yezo virus strain T-HLJ02 (ON563272.1) | 100.0% | 99.0% |
| Nairoviridae | Orthonairovirus | K_YZV | | 1620 | 0.07 | Yezo virus strain T-HLJ02 (ON563273.1) | 100.0% | 98.6% |
| Partitiviridae | Deltapartitivirus-like | D_JLPV1 | | 1714 | 1.50 | Jilin partiti-like virus 1 strain MDJ2 (ON408189.1) | 99.0% | 99.7% |
| Phenuiviridae | Phlebovirus | D_MKWV | | 6486 | 4.99 | Mukawa virus strain NE-TH3 segment L (ON408114.1) | 98.0% | 98.5% |
| Phenuiviridae | Phlebovirus | D_MKWV | | 3124 | 3.18 | Mukawa virus strain XQ1 segment M (OR730566.1) | 99.0% | 97.3% |
| Phenuiviridae | Phlebovirus | D_MKWV | | 1960 | 2.52 | Mukawa virus strain NE-TH3 segment S (ON408116.1) | 95.0% | 98.4% |
| Phenuiviridae | lxovirus | D_OTPV | | 3674 | 1.90 | Onega tick phlebovirus strain NE-SL3 segment L (ON408154.1) | 99.0% | 99.6% |
| Phenuiviridae | lxovirus | D_OTPV | | 1921 | 2.36 | Onega tick phlebovirus strain NE-SL3 segment S (ON408155.1) | 100.0% | 100.0% |
| Phenuiviridae | lxovirus | D_STPV | | 4757 | 3.42 | Sara tick phlebovirus strain NE-SL4 segment L (ON408144.1) | 99.0% | 99.7% |
| Phenuiviridae | lxovirus | D_STPV | | 2483 | 3.20 | Sara tick phlebovirus strain NE-SL3 segment S (ON408143.1) | 100.0% | 99.8% |
| Phenuiviridae | Phlebovirus | K_MKWV | | 6446 | 2.66 | Mukawa virus strain YKS1 segment L (OR730562.1) | 99.0% | 89.1% |
| Phenuiviridae | Phlebovirus | K_MKWV | | 3219 | 2.74 | Mukawa virus strain NE-TH3 segment M (ON408115.1) | 99.0% | 98.8% |
| Phenuiviridae | lxovirus | K_OTPV | | 6662 | 1.62 | Onega tick phlebovirus strain NE-SL3 segment L (ON408154.1) | 100.0% | 99.6% |
| Phenuiviridae | lxovirus | K_OTPV | | 1921 | 2.24 | Onega tick phlebovirus strain NE-TH4 segment S (ON408153.1) | 99.0% | 100.0% |
| Phenuiviridae | lxovirus | K_STPV | | 6679 | 2.74 | Sara tick phlebovirus strain NE-TH3 segment L (ON408138.1) | 99.0% | 99.6% |
| Phenuiviridae | lxovirus | K_STPV | | 2470 | 5.19 | Sara tick phlebovirus strain NE-SL4 segment S (ON408145.1) | 91.0% | 100.0% |
| Rhabdoviridae | Alphanemrhavirus-like | D_THRV2 | | 11567 | 4.55 | Tahe rhabdovirus 2 strain NE-TH4 (ON408171.1) | 99.0% | 98.7% |
| Rhabdoviridae | Alphanemrhavirus-like | D_THRV3 | | 5324 | 2.84 | Tahe rhabdovirus 3 strain NE-TH3 ( ON408172.1) | 99.0% | 99.9% |
| Rhabdoviridae | Alphanemrhavirus-like | G_THRV1 | | 11362 | 1.74 | Tahe rhabdovirus 1 strain NE-SL1 ( ON408164.1) | 99.0% | 99.6% |
| Rhabdoviridae | Alphanemrhavirus-like | K_THRV2 | | 11488 | 1.58 | Tahe rhabdovirus 2 strain NE-TH3 (ON408170.1) | 99.0% | 99.2% |
| Rhabdoviridae | Alphanemrhavirus-like | K_THRV3 | | 10365 | 2.15 | Tahe rhabdovirus 3 strain NE-TH3 (ON408172.1) | 99.0% | 99.7% |
| Solemoviridae | Sobemo-like | B_XTAV1 | | 2588 | 0.78 | Xinjiang tick associated virus 1 isolate NM-DS-18 (OP863284.1) | 99.0% | 98.9% |
| Solemoviridae | Sobemo-like | D_ISAV1 | | 2654 | 1.68 | Ixodes scapularis associated virus 1 strain YC3 (ON408199.1) | 99.0% | 98.5% |
| Solemoviridae | Sobemo-like | D_JLLV2 | | 1120 | 2.74 | Jilin luteo-like virus 2 strain DH3 (ON408211.1) | 100.0% | 98.9% |
| Solemoviridae | Sobemo-like | K_ISAV1 | | 2603 | 1.17 | Ixodes scapularis associated virus 1 strain YC3 (ON408199.1) | 99.0% | 99.2% |
| Totiviridae | unclassified Totiviridae | B_Totiviridae sp. | | 9022 | 1.47 | Totiviridae sp. isolate TIGMIC_1 (ON812582.1) | 99.0% | 96.9% |
| Totiviridae | unclassified Totiviridae | G_Totiviridae sp. | | 5706 | 0.73 | Totiviridae sp. isolate TIGMIC_10 (ON812602.1) | 100.0% | 98.6% |
