# Supplementary material for: Metatranscriptomic analysis reveals the diversity of RNA viruses in ticks in Inner Mongolia, China
Source: PLoS Negl Trop Dis. 2024 Dec 11;18(12):e0012706. doi: 10.1371/journal.pntd.0012706 (PMC11634002; doi:10.1371/journal.pntd.0012706)
Supplement: S3 Table — (DOC) [file pntd.0012706.s003.doc]

**S3 Table. Primer sequences used in virus detection.**

| **Family** | **Virus name** | **Primer sequence** | **Annealing temperature(℃)** | **Amplication size(bp)** |
| --- | --- | --- | --- | --- |
| Phenuiviridae | Onega tick phlebovirus | ATTTCTGGCTTATTGCGTTG | 54 | 499 |
|  |  | ACAGTTCTTTCGTGCGTTC | 56 |  |
|  | Sara tick phlebovirus | TGCGACATGATTACACTGCT | 57 | 352 |
|  |  | ACCAAGAACCTGACGCAAA | 57 |  |
|  | Mukawa virus | TGCTCCATGTTCACGAA | 53 | 425 |
|  |  | AGCCTCTTAATTCTAAAGCAA | 52 |  |
| Chuviridae | Bole tick virus 3 | TGGTCATATTCACCTCTCCC | 56 | 413 |
|  |  | TGAAGCAGAAGGTATTTGGC | 56 |  |
|  | Chuviridae sp. | CGTCAACCTCTTCCAACGCTA | 57 | 619 |
|  |  | GACTGGAAAGAAACCCGACT | 60 |  |
|  | Taiga tick nigecruvirus | CTGCTGCTCTTTTACTTGCT | 55 | 268 |
|  |  | GGGCAATTTCACCTAATGTCA | 56 |  |
|  | Nuomin virus | GCATAATCTTTGTACTCCGTCA | 56 | 203 |
|  |  | TGTGATGCTTCTTCCTCGT | 56 |  |
| Nairoviridae | Yezo virus | GAGGAAGTTAGGATGGAGCT | 56 | 423 |
|  |  | ATGGACAAGTAGACCGCTAA | 56 |  |
|  | Beiji nairovirus | CAGACTGCACTTATGAAGCC | 56 | 216 |
|  |  | TTTTCCCACAGAAAGCGAGA | 57 |  |
| Flaviviridae | Bole tick virus 4 | CCGACAGAGTTGATGACATC | 56 | 362 |
|  |  | GCTGAGAATGGTAATTGCCA | 56 |  |
|  | Bole tick virus 4 (2) | TCCACAACAAAGTCGAATCC | 56 | 331 |
|  |  | TCGTAGAAACTGGAGACGAA | 56 |  |
|  | Tick-borne encephalitis virus | GTTCAATGATCTGGCTCTACCG | 58 | 340 |
|  |  | GAACGTGACCTCCATAACCAC | 58 |  |
|  | Alongshan virus | TACAACGTGGCCTTACACC | 57 | 311 |
|  |  | TATCACCAGGAGCTTTACGG | 56 |  |
| Rhabdoviridae | Tahe rhabdovirus 1 | AGACTCCGAACAGCTATCAA | 56 | 385 |
|  |  | CTTCAATCTCGCGTACACAT | 56 |  |
|  | Tahe rhabdovirus 2 | ACATCCGAGCCCTATATCTG | 56 | 342 |
|  |  | GCTTCCGAAAAGAGAGTGTT | 56 |  |
|  | Tahe rhabdovirus 3 | TATTACCGTCTTGACATTGACC | 55 | 296 |
|  |  | CTCCCTTCTAGTTTCCGTTG | 55 |  |
| Solemoviridae | Xinjiang tick associated virus 1 | GCTTTCGTTTCTTTGCTACG | 56 | 260 |
|  |  | GTAAAAAGCCAAATAGCCGC | 56 |  |
|  | Ixodes scapularis associated virus 1 | CCGATCCTGCTTTATGTCGT | 57 | 205 |
|  |  | AGAAGCACAAACCGTACCAC | 58 |  |
|  | Jilin luteo-like virus 2 | GGTTCTCGTCAAGCCCAA | 57 | 275 |
|  |  | TTCATTTCGCCTAACCATC | 53 |  |
| Partitiviridae | Jilin partiti-like virus 1 | TCCTGGATCTCTTGAGTTCC | 56 | 427 |
|  |  | ATCCCTTCGTGTCCTTTTTG | 56 |  |
| Totiviridae | Totiviridae sp. | GCCATACCAAAGGTCTTGTT | 56 | 328 |
|  |  | AGATTGTCTTCGTTCGCAAA | 56 |  |
| Bunyavirales_norank | Volzhskoe tick virus | CTCAGGTGTTGTTCTCACTG | 56 | 317 |
|  |  | AGGTTATCTCGGTTTTTGCC | 56 |  |
