# Supplementary material for: Metatranscriptomic analysis reveals the diversity of RNA viruses in ticks in Inner Mongolia, China
Source: PLoS Negl Trop Dis. 2024 Dec 11;18(12):e0012706. doi: 10.1371/journal.pntd.0012706 (PMC11634002; doi:10.1371/journal.pntd.0012706)
Supplement: S4 Table — (DOC) [file pntd.0012706.s004.doc]

**S4 Table. Accession numbers of viral sequences amplified by PCR.**

| BankIt ID | Pools name | Collection sites | Host species | GenBank accession NO. | Viral species |
| --- | --- | --- | --- | --- | --- |
| BankIt2735143 | D8 | Hinggan League | Ixodes persulcatus | OR454143 | Beiji nairovirus |
| BankIt2735143 | D16 | Hinggan League | Ixodes persulcatus | OR454144 | Beiji nairovirus |
| BankIt2735143 | D12 | Hinggan League | Ixodes persulcatus | OR454145 | Beiji nairovirus |
| BankIt2735143 | D7 | Hinggan League | Ixodes persulcatus | OR454146 | Beiji nairovirus |
| BankIt2735143 | D1 | Hinggan League | Ixodes persulcatus | OR454147 | Beiji nairovirus |
| BankIt2735143 | D4 | Hinggan League | Ixodes persulcatus | OR454148 | Beiji nairovirus |
| BankIt2735143 | D20 | Hinggan League | Ixodes persulcatus | OR454149 | Beiji nairovirus |
| BankIt2735143 | D10 | Hinggan League | Ixodes persulcatus | OR454150 | Beiji nairovirus |
| BankIt2735143 | D13 | Hinggan League | Ixodes persulcatus | OR454151 | Beiji nairovirus |
| BankIt2735143 | D25 | Hinggan League | Ixodes persulcatus | OR454152 | Beiji nairovirus |
| BankIt2735143 | D19 | Hinggan League | Ixodes persulcatus | OR454153 | Beiji nairovirus |
| BankIt2735143 | D15 | Hinggan League | Ixodes persulcatus | OR454154 | Beiji nairovirus |
| BankIt2735143 | D3 | Hinggan League | Ixodes persulcatus | OR454155 | Beiji nairovirus |
| BankIt2735143 | D14 | Hinggan League | Ixodes persulcatus | OR454156 | Beiji nairovirus |
| BankIt2735143 | D24 | Hinggan League | Ixodes persulcatus | OR454157 | Beiji nairovirus |
| BankIt2735143 | D17 | Hinggan League | Ixodes persulcatus | OR454158 | Beiji nairovirus |
| BankIt2735143 | D21 | Hinggan League | Ixodes persulcatus | OR454159 | Beiji nairovirus |
| BankIt2735143 | D22 | Hinggan League | Ixodes persulcatus | OR454160 | Beiji nairovirus |
| BankIt2735143 | D6 | Hinggan League | Ixodes persulcatus | OR454161 | Beiji nairovirus |
| BankIt2735143 | D27 | Hinggan League | Ixodes persulcatus | OR454162 | Beiji nairovirus |
| BankIt2735143 | D18 | Hinggan League | Ixodes persulcatus | OR454163 | Beiji nairovirus |
| BankIt2735143 | D23 | Hinggan League | Ixodes persulcatus | OR454164 | Beiji nairovirus |
| BankIt2735143 | D11 | Hinggan League | Ixodes persulcatus | OR454165 | Beiji nairovirus |
| BankIt2756831 | K10 | Hulun Buir | Ixodes persulcatus | OR723930 | [Beiji nairovirus](https://www.ncbi.nlm.nih.gov/Taxonomy/Browser/wwwtax.cgi?id=2304647) |
| BankIt2756831 | K5 | Hulun Buir | Ixodes persulcatus | OR723931 | [Beiji nairovirus](https://www.ncbi.nlm.nih.gov/Taxonomy/Browser/wwwtax.cgi?id=2304647) |
| BankIt2756831 | K2 | Hulun Buir | Ixodes persulcatus | OR723932 | [Beiji nairovirus](https://www.ncbi.nlm.nih.gov/Taxonomy/Browser/wwwtax.cgi?id=2304647) |
| BankIt2756831 | K11 | Hulun Buir | Ixodes persulcatus | OR723933 | [Beiji nairovirus](https://www.ncbi.nlm.nih.gov/Taxonomy/Browser/wwwtax.cgi?id=2304647) |
| BankIt2756831 | K6 | Hulun Buir | Ixodes persulcatus | OR723934 | [Beiji nairovirus](https://www.ncbi.nlm.nih.gov/Taxonomy/Browser/wwwtax.cgi?id=2304647) |
| BankIt2756831 | K3 | Hulun Buir | Ixodes persulcatus | OR723935 | [Beiji nairovirus](https://www.ncbi.nlm.nih.gov/Taxonomy/Browser/wwwtax.cgi?id=2304647) |
| BankIt2756831 | K12 | Hulun Buir | Ixodes persulcatus | OR723936 | [Beiji nairovirus](https://www.ncbi.nlm.nih.gov/Taxonomy/Browser/wwwtax.cgi?id=2304647) |
| BankIt2756831 | K7 | Hulun Buir | Ixodes persulcatus | OR723937 | [Beiji nairovirus](https://www.ncbi.nlm.nih.gov/Taxonomy/Browser/wwwtax.cgi?id=2304647) |
| BankIt2756831 | K13 | Hulun Buir | Ixodes persulcatus | OR723938 | [Beiji nairovirus](https://www.ncbi.nlm.nih.gov/Taxonomy/Browser/wwwtax.cgi?id=2304647) |
| BankIt2756831 | K8 | Hulun Buir | Ixodes persulcatus | OR723939 | [Beiji nairovirus](https://www.ncbi.nlm.nih.gov/Taxonomy/Browser/wwwtax.cgi?id=2304647) |
| BankIt2756831 | K14 | Hulun Buir | Ixodes persulcatus | OR723940 | [Beiji nairovirus](https://www.ncbi.nlm.nih.gov/Taxonomy/Browser/wwwtax.cgi?id=2304647) |
| BankIt2756831 | K9 | Hulun Buir | Ixodes persulcatus | OR723941 | [Beiji nairovirus](https://www.ncbi.nlm.nih.gov/Taxonomy/Browser/wwwtax.cgi?id=2304647) |
| BankIt2756965 | D1 | Hinggan League | Ixodes persulcatus | OR723942 | [Sara tick phlebovirus](https://blast.ncbi.nlm.nih.gov/Blast.cgi#alnHdr_2314145054) |
| BankIt2756965 | D19 | Hinggan League | Ixodes persulcatus | OR723943 | [Sara tick phlebovirus](https://blast.ncbi.nlm.nih.gov/Blast.cgi#alnHdr_2314145054) |
| BankIt2756965 | D3 | Hinggan League | Ixodes persulcatus | OR723944 | [Sara tick phlebovirus](https://blast.ncbi.nlm.nih.gov/Blast.cgi#alnHdr_2314145054) |
| BankIt2756965 | D2 | Hinggan League | Ixodes persulcatus | OR723945 | [Sara tick phlebovirus](https://blast.ncbi.nlm.nih.gov/Blast.cgi#alnHdr_2314145054) |
| BankIt2756965 | D4 | Hinggan League | Ixodes persulcatus | OR723946 | [Sara tick phlebovirus](https://blast.ncbi.nlm.nih.gov/Blast.cgi#alnHdr_2314145054) |
| BankIt2756965 | D8 | Hinggan League | Ixodes persulcatus | OR723947 | [Sara tick phlebovirus](https://blast.ncbi.nlm.nih.gov/Blast.cgi#alnHdr_2314145054) |
| BankIt2756965 | D9 | Hinggan League | Ixodes persulcatus | OR723948 | [Sara tick phlebovirus](https://blast.ncbi.nlm.nih.gov/Blast.cgi#alnHdr_2314145054) |
| BankIt2756965 | D11 | Hinggan League | Ixodes persulcatus | OR723949 | [Sara tick phlebovirus](https://blast.ncbi.nlm.nih.gov/Blast.cgi#alnHdr_2314145054) |
| BankIt2756965 | D12 | Hinggan League | Ixodes persulcatus | OR723950 | [Sara tick phlebovirus](https://blast.ncbi.nlm.nih.gov/Blast.cgi#alnHdr_2314145054) |
| BankIt2756965 | D13 | Hinggan League | Ixodes persulcatus | OR723951 | [Sara tick phlebovirus](https://blast.ncbi.nlm.nih.gov/Blast.cgi#alnHdr_2314145054) |
| BankIt2756965 | D15 | Hinggan League | Ixodes persulcatus | OR723952 | [Sara tick phlebovirus](https://blast.ncbi.nlm.nih.gov/Blast.cgi#alnHdr_2314145054) |
| BankIt2756965 | D16 | Hinggan League | Ixodes persulcatus | OR723953 | [Sara tick phlebovirus](https://blast.ncbi.nlm.nih.gov/Blast.cgi#alnHdr_2314145054) |
| BankIt2756965 | D17 | Hinggan League | Ixodes persulcatus | OR723954 | [Sara tick phlebovirus](https://blast.ncbi.nlm.nih.gov/Blast.cgi#alnHdr_2314145054) |
| BankIt2756965 | D18 | Hinggan League | Ixodes persulcatus | OR723955 | [Sara tick phlebovirus](https://blast.ncbi.nlm.nih.gov/Blast.cgi#alnHdr_2314145054) |
| BankIt2756965 | D21 | Hinggan League | Ixodes persulcatus | OR723956 | [Sara tick phlebovirus](https://blast.ncbi.nlm.nih.gov/Blast.cgi#alnHdr_2314145054) |
| BankIt2756965 | D22 | Hinggan League | Ixodes persulcatus | OR723957 | [Sara tick phlebovirus](https://blast.ncbi.nlm.nih.gov/Blast.cgi#alnHdr_2314145054) |
| BankIt2756965 | D23 | Hinggan League | Ixodes persulcatus | OR723958 | [Sara tick phlebovirus](https://blast.ncbi.nlm.nih.gov/Blast.cgi#alnHdr_2314145054) |
| BankIt2756965 | D24 | Hinggan League | Ixodes persulcatus | OR723959 | [Sara tick phlebovirus](https://blast.ncbi.nlm.nih.gov/Blast.cgi#alnHdr_2314145054) |
| BankIt2756965 | D25 | Hinggan League | Ixodes persulcatus | OR723960 | [Sara tick phlebovirus](https://blast.ncbi.nlm.nih.gov/Blast.cgi#alnHdr_2314145054) |
| BankIt2756965 | D26 | Hinggan League | Ixodes persulcatus | OR723961 | [Sara tick phlebovirus](https://blast.ncbi.nlm.nih.gov/Blast.cgi#alnHdr_2314145054) |
| BankIt2756965 | K9 | Hulun Buir | Ixodes persulcatus | OR723962 | [Sara tick phlebovirus](https://blast.ncbi.nlm.nih.gov/Blast.cgi#alnHdr_2314145054) |
| BankIt2756965 | K10 | Hulun Buir | Ixodes persulcatus | OR723963 | [Sara tick phlebovirus](https://blast.ncbi.nlm.nih.gov/Blast.cgi#alnHdr_2314145054) |
| BankIt2756965 | K11 | Hulun Buir | Ixodes persulcatus | OR723964 | [Sara tick phlebovirus](https://blast.ncbi.nlm.nih.gov/Blast.cgi#alnHdr_2314145054) |
| BankIt2756965 | K12 | Hulun Buir | Ixodes persulcatus | OR723965 | [Sara tick phlebovirus](https://blast.ncbi.nlm.nih.gov/Blast.cgi#alnHdr_2314145054) |
| BankIt2756965 | K13 | Hulun Buir | Ixodes persulcatus | OR723966 | [Sara tick phlebovirus](https://blast.ncbi.nlm.nih.gov/Blast.cgi#alnHdr_2314145054) |
| BankIt2756965 | K14 | Hulun Buir | Ixodes persulcatus | OR723967 | [Sara tick phlebovirus](https://blast.ncbi.nlm.nih.gov/Blast.cgi#alnHdr_2314145054) |
| BankIt2757875 | D1 | Hinggan League | Ixodes persulcatus | OR737808 | [Mukawa virus](https://www.ncbi.nlm.nih.gov/Taxonomy/Browser/wwwtax.cgi?id=1569922) |
| BankIt2757875 | D7 | Hinggan League | Ixodes persulcatus | OR737809 | [Mukawa virus](https://www.ncbi.nlm.nih.gov/Taxonomy/Browser/wwwtax.cgi?id=1569922) |
| BankIt2757875 | D4 | Hinggan League | Ixodes persulcatus | OR737810 | [Mukawa virus](https://www.ncbi.nlm.nih.gov/Taxonomy/Browser/wwwtax.cgi?id=1569922) |
| BankIt2757875 | D2 | Hinggan League | Ixodes persulcatus | OR737811 | [Mukawa virus](https://www.ncbi.nlm.nih.gov/Taxonomy/Browser/wwwtax.cgi?id=1569922) |
| BankIt2757875 | D6 | Hinggan League | Ixodes persulcatus | OR737812 | [Mukawa virus](https://www.ncbi.nlm.nih.gov/Taxonomy/Browser/wwwtax.cgi?id=1569922) |
| BankIt2757875 | D18 | Hinggan League | Ixodes persulcatus | OR737813 | [Mukawa virus](https://www.ncbi.nlm.nih.gov/Taxonomy/Browser/wwwtax.cgi?id=1569922) |
| BankIt2757875 | D25 | Hinggan League | Ixodes persulcatus | OR737814 | [Mukawa virus](https://www.ncbi.nlm.nih.gov/Taxonomy/Browser/wwwtax.cgi?id=1569922) |
| BankIt2757875 | D27 | Hinggan League | Ixodes persulcatus | OR737815 | [Mukawa virus](https://www.ncbi.nlm.nih.gov/Taxonomy/Browser/wwwtax.cgi?id=1569922) |
| BankIt2758170 | K4 | Hulun Buir | Ixodes persulcatus | OR737816 | [Alongshan virus](https://www.ncbi.nlm.nih.gov/Taxonomy/Browser/wwwtax.cgi?id=2269360) |
| BankIt2758170 | K9 | Hulun Buir | Ixodes persulcatus | OR737817 | [Alongshan virus](https://www.ncbi.nlm.nih.gov/Taxonomy/Browser/wwwtax.cgi?id=2269360) |
| BankIt2758170 | K14 | Hulun Buir | Ixodes persulcatus | OR737818 | [Alongshan virus](https://www.ncbi.nlm.nih.gov/Taxonomy/Browser/wwwtax.cgi?id=2269360) |
| BankIt2758170 | D1 | Hinggan League | Ixodes persulcatus | OR737819 | [Alongshan virus](https://www.ncbi.nlm.nih.gov/Taxonomy/Browser/wwwtax.cgi?id=2269360) |
| BankIt2758170 | D2 | Hinggan League | Ixodes persulcatus | OR737820 | [Alongshan virus](https://www.ncbi.nlm.nih.gov/Taxonomy/Browser/wwwtax.cgi?id=2269360) |
| BankIt2758170 | D3 | Hinggan League | Ixodes persulcatus | OR737821 | [Alongshan virus](https://www.ncbi.nlm.nih.gov/Taxonomy/Browser/wwwtax.cgi?id=2269360) |
| BankIt2758170 | D4 | Hinggan League | Ixodes persulcatus | OR737822 | [Alongshan virus](https://www.ncbi.nlm.nih.gov/Taxonomy/Browser/wwwtax.cgi?id=2269360) |
| BankIt2758170 | D8 | Hinggan League | Ixodes persulcatus | OR737823 | [Alongshan virus](https://www.ncbi.nlm.nih.gov/Taxonomy/Browser/wwwtax.cgi?id=2269360) |
| BankIt2758170 | D20 | Hinggan League | Ixodes persulcatus | OR737824 | [Alongshan virus](https://www.ncbi.nlm.nih.gov/Taxonomy/Browser/wwwtax.cgi?id=2269360) |
| BankIt2758170 | D23 | Hinggan League | Ixodes persulcatus | OR737825 | [Alongshan virus](https://www.ncbi.nlm.nih.gov/Taxonomy/Browser/wwwtax.cgi?id=2269360) |
| BankIt2758184 | D2 | Hinggan League | Ixodes persulcatus | OR737826 | [Nuomin virus](https://www.ncbi.nlm.nih.gov/Taxonomy/Browser/wwwtax.cgi?id=2916986) |
| BankIt2758184 | D1 | Hinggan League | Ixodes persulcatus | OR737827 | [Nuomin virus](https://www.ncbi.nlm.nih.gov/Taxonomy/Browser/wwwtax.cgi?id=2916986) |
| BankIt2758184 | D12 | Hinggan League | Ixodes persulcatus | OR737828 | [Nuomin virus](https://www.ncbi.nlm.nih.gov/Taxonomy/Browser/wwwtax.cgi?id=2916986) |
| BankIt2758184 | D4 | Hinggan League | Ixodes persulcatus | OR737829 | [Nuomin virus](https://www.ncbi.nlm.nih.gov/Taxonomy/Browser/wwwtax.cgi?id=2916986) |
| BankIt2758184 | D11 | Hinggan League | Ixodes persulcatus | OR737830 | [Nuomin virus](https://www.ncbi.nlm.nih.gov/Taxonomy/Browser/wwwtax.cgi?id=2916986) |
| BankIt2758184 | D15 | Hinggan League | Ixodes persulcatus | OR737831 | [Nuomin virus](https://www.ncbi.nlm.nih.gov/Taxonomy/Browser/wwwtax.cgi?id=2916986) |
| BankIt2758184 | D9 | Hinggan League | Ixodes persulcatus | OR737832 | [Nuomin virus](https://www.ncbi.nlm.nih.gov/Taxonomy/Browser/wwwtax.cgi?id=2916986) |
| BankIt2758184 | D16 | Hinggan League | Ixodes persulcatus | OR737833 | [Nuomin virus](https://www.ncbi.nlm.nih.gov/Taxonomy/Browser/wwwtax.cgi?id=2916986) |
| BankIt2758184 | D13 | Hinggan League | Ixodes persulcatus | OR737834 | [Nuomin virus](https://www.ncbi.nlm.nih.gov/Taxonomy/Browser/wwwtax.cgi?id=2916986) |
| BankIt2758184 | D18 | Hinggan League | Ixodes persulcatus | OR737835 | [Nuomin virus](https://www.ncbi.nlm.nih.gov/Taxonomy/Browser/wwwtax.cgi?id=2916986) |
| BankIt2758184 | D20 | Hinggan League | Ixodes persulcatus | OR737836 | [Nuomin virus](https://www.ncbi.nlm.nih.gov/Taxonomy/Browser/wwwtax.cgi?id=2916986) |
| BankIt2758184 | D21 | Hinggan League | Ixodes persulcatus | OR737837 | [Nuomin virus](https://www.ncbi.nlm.nih.gov/Taxonomy/Browser/wwwtax.cgi?id=2916986) |
| BankIt2758184 | D23 | Hinggan League | Ixodes persulcatus | OR737838 | [Nuomin virus](https://www.ncbi.nlm.nih.gov/Taxonomy/Browser/wwwtax.cgi?id=2916986) |
| BankIt2758184 | D25 | Hinggan League | Ixodes persulcatus | OR737839 | [Nuomin virus](https://www.ncbi.nlm.nih.gov/Taxonomy/Browser/wwwtax.cgi?id=2916986) |
| BankIt2758184 | D14 | Hinggan League | Ixodes persulcatus | OR737840 | [Nuomin virus](https://www.ncbi.nlm.nih.gov/Taxonomy/Browser/wwwtax.cgi?id=2916986) |
| BankIt2758184 | D6 | Hinggan League | Ixodes persulcatus | OR737841 | [Nuomin virus](https://www.ncbi.nlm.nih.gov/Taxonomy/Browser/wwwtax.cgi?id=2916986) |
| BankIt2758184 | D27 | Hinggan League | Ixodes persulcatus | OR737842 | [Nuomin virus](https://www.ncbi.nlm.nih.gov/Taxonomy/Browser/wwwtax.cgi?id=2916986) |
| BankIt2758184 | K12 | Hulun Buir | Ixodes persulcatus | OR737843 | [Nuomin virus](https://www.ncbi.nlm.nih.gov/Taxonomy/Browser/wwwtax.cgi?id=2916986) |
| BankIt2758184 | K14 | Hulun Buir | Ixodes persulcatus | OR737844 | [Nuomin virus](https://www.ncbi.nlm.nih.gov/Taxonomy/Browser/wwwtax.cgi?id=2916986) |
| BankIt2758316 | D7 | Hinggan League | Ixodes persulcatus | OR744750 | [Tahe rhabdovirus 3](https://www.ncbi.nlm.nih.gov/Taxonomy/Browser/wwwtax.cgi?id=2983976) |
| BankIt2758316 | D1 | Hinggan League | Ixodes persulcatus | OR744751 | [Tahe rhabdovirus 3](https://www.ncbi.nlm.nih.gov/Taxonomy/Browser/wwwtax.cgi?id=2983976) |
| BankIt2758316 | D12 | Hinggan League | Ixodes persulcatus | OR744752 | [Tahe rhabdovirus 3](https://www.ncbi.nlm.nih.gov/Taxonomy/Browser/wwwtax.cgi?id=2983976) |
| BankIt2758316 | D16 | Hinggan League | Ixodes persulcatus | OR744753 | [Tahe rhabdovirus 3](https://www.ncbi.nlm.nih.gov/Taxonomy/Browser/wwwtax.cgi?id=2983976) |
| BankIt2758316 | D8 | Hinggan League | Ixodes persulcatus | OR744754 | [Tahe rhabdovirus 3](https://www.ncbi.nlm.nih.gov/Taxonomy/Browser/wwwtax.cgi?id=2983976) |
| BankIt2758316 | D26 | Hinggan League | Ixodes persulcatus | OR744755 | [Tahe rhabdovirus 3](https://www.ncbi.nlm.nih.gov/Taxonomy/Browser/wwwtax.cgi?id=2983976) |
| BankIt2758316 | D2 | Hinggan League | Ixodes persulcatus | OR744756 | [Tahe rhabdovirus 3](https://www.ncbi.nlm.nih.gov/Taxonomy/Browser/wwwtax.cgi?id=2983976) |
| BankIt2758316 | D13 | Hinggan League | Ixodes persulcatus | OR744757 | [Tahe rhabdovirus 3](https://www.ncbi.nlm.nih.gov/Taxonomy/Browser/wwwtax.cgi?id=2983976) |
| BankIt2758316 | D10 | Hinggan League | Ixodes persulcatus | OR744758 | [Tahe rhabdovirus 3](https://www.ncbi.nlm.nih.gov/Taxonomy/Browser/wwwtax.cgi?id=2983976) |
| BankIt2758316 | D3 | Hinggan League | Ixodes persulcatus | OR744759 | [Tahe rhabdovirus 3](https://www.ncbi.nlm.nih.gov/Taxonomy/Browser/wwwtax.cgi?id=2983976) |
| BankIt2758316 | D14 | Hinggan League | Ixodes persulcatus | OR744760 | [Tahe rhabdovirus 3](https://www.ncbi.nlm.nih.gov/Taxonomy/Browser/wwwtax.cgi?id=2983976) |
| BankIt2758316 | D21 | Hinggan League | Ixodes persulcatus | OR744761 | [Tahe rhabdovirus 3](https://www.ncbi.nlm.nih.gov/Taxonomy/Browser/wwwtax.cgi?id=2983976) |
| BankIt2758316 | D22 | Hinggan League | Ixodes persulcatus | OR744762 | [Tahe rhabdovirus 3](https://www.ncbi.nlm.nih.gov/Taxonomy/Browser/wwwtax.cgi?id=2983976) |
| BankIt2758316 | K11 | Hulun Buir | Ixodes persulcatus | OR744763 | [Tahe rhabdovirus 3](https://www.ncbi.nlm.nih.gov/Taxonomy/Browser/wwwtax.cgi?id=2983976) |
| BankIt2758399 | E48 | Bayan Nur | Hyalomma marginatum | OR744764 | [Bole tick virus 4](https://www.ncbi.nlm.nih.gov/Taxonomy/Browser/wwwtax.cgi?id=1746058) |
| BankIt2758399 | E4 | Bayan Nur | Hyalomma marginatum | OR744765 | [Bole tick virus 4](https://www.ncbi.nlm.nih.gov/Taxonomy/Browser/wwwtax.cgi?id=1746058) |
| BankIt2758399 | E12 | Bayan Nur | Hyalomma marginatum | OR744766 | [Bole tick virus 4](https://www.ncbi.nlm.nih.gov/Taxonomy/Browser/wwwtax.cgi?id=1746058) |
| BankIt2758399 | E6 | Bayan Nur | Hyalomma marginatum | OR744767 | [Bole tick virus 4](https://www.ncbi.nlm.nih.gov/Taxonomy/Browser/wwwtax.cgi?id=1746058) |
| BankIt2758399 | E19 | Bayan Nur | Hyalomma marginatum | OR744768 | [Bole tick virus 4](https://www.ncbi.nlm.nih.gov/Taxonomy/Browser/wwwtax.cgi?id=1746058) |
| BankIt2758399 | E8 | Bayan Nur | Hyalomma marginatum | OR744769 | [Bole tick virus 4](https://www.ncbi.nlm.nih.gov/Taxonomy/Browser/wwwtax.cgi?id=1746058) |
| BankIt2758399 | E21 | Bayan Nur | Hyalomma marginatum | OR744770 | [Bole tick virus 4](https://www.ncbi.nlm.nih.gov/Taxonomy/Browser/wwwtax.cgi?id=1746058) |
| BankIt2758399 | E11 | Bayan Nur | Hyalomma marginatum | OR744771 | [Bole tick virus 4](https://www.ncbi.nlm.nih.gov/Taxonomy/Browser/wwwtax.cgi?id=1746058) |
| BankIt2758399 | E24 | Bayan Nur | Hyalomma marginatum | OR744772 | [Bole tick virus 4](https://www.ncbi.nlm.nih.gov/Taxonomy/Browser/wwwtax.cgi?id=1746058) |
| BankIt2758399 | E26 | Bayan Nur | Hyalomma marginatum | OR744773 | [Bole tick virus 4](https://www.ncbi.nlm.nih.gov/Taxonomy/Browser/wwwtax.cgi?id=1746058) |
| BankIt2758399 | E30 | Bayan Nur | Hyalomma marginatum | OR744774 | [Bole tick virus 4](https://www.ncbi.nlm.nih.gov/Taxonomy/Browser/wwwtax.cgi?id=1746058) |
| BankIt2758399 | E32 | Bayan Nur | Hyalomma marginatum | OR744775 | [Bole tick virus 4](https://www.ncbi.nlm.nih.gov/Taxonomy/Browser/wwwtax.cgi?id=1746058) |
| BankIt2758399 | E36 | Bayan Nur | Hyalomma marginatum | OR744776 | [Bole tick virus 4](https://www.ncbi.nlm.nih.gov/Taxonomy/Browser/wwwtax.cgi?id=1746058) |
| BankIt2758399 | E38 | Bayan Nur | Hyalomma marginatum | OR744777 | [Bole tick virus 4](https://www.ncbi.nlm.nih.gov/Taxonomy/Browser/wwwtax.cgi?id=1746058) |
| BankIt2758399 | E39 | Bayan Nur | Hyalomma marginatum | OR744778 | [Bole tick virus 4](https://www.ncbi.nlm.nih.gov/Taxonomy/Browser/wwwtax.cgi?id=1746058) |
| BankIt2758399 | E40 | Bayan Nur | Hyalomma marginatum | OR744779 | [Bole tick virus 4](https://www.ncbi.nlm.nih.gov/Taxonomy/Browser/wwwtax.cgi?id=1746058) |
| BankIt2758399 | E41 | Bayan Nur | Hyalomma marginatum | OR744780 | [Bole tick virus 4](https://www.ncbi.nlm.nih.gov/Taxonomy/Browser/wwwtax.cgi?id=1746058) |
| BankIt2758399 | E42 | Bayan Nur | Hyalomma marginatum | OR744781 | [Bole tick virus 4](https://www.ncbi.nlm.nih.gov/Taxonomy/Browser/wwwtax.cgi?id=1746058) |
| BankIt2758399 | E43 | Bayan Nur | Hyalomma marginatum | OR744782 | [Bole tick virus 4](https://www.ncbi.nlm.nih.gov/Taxonomy/Browser/wwwtax.cgi?id=1746058) |
| BankIt2758399 | E47 | Bayan Nur | Hyalomma marginatum | OR744783 | [Bole tick virus 4](https://www.ncbi.nlm.nih.gov/Taxonomy/Browser/wwwtax.cgi?id=1746058) |
| BankIt2758542 | B4 | Bayan Nur | Dermacentor nuttalli | OR744784 | Bole tick virus 4 |
| BankIt2758542 | B21 | Bayan Nur | Dermacentor nuttalli | OR744785 | Bole tick virus 4 |
| BankIt2758542 | B20 | Bayan Nur | Dermacentor nuttalli | OR744786 | Bole tick virus 4 |
| BankIt2758542 | B19 | Bayan Nur | Dermacentor nuttalli | OR744787 | Bole tick virus 4 |
| BankIt2758542 | B13 | Bayan Nur | Dermacentor nuttalli | OR744788 | Bole tick virus 4 |
| BankIt2758542 | B12 | Bayan Nur | Dermacentor nuttalli | OR744789 | Bole tick virus 4 |
| BankIt2758593 | D1 | Hinggan League | Ixodes persulcatus | OR744790 | [Tick-borne encephalitis virus](https://www.ncbi.nlm.nih.gov/Taxonomy/Browser/wwwtax.cgi?id=11084) |
| BankIt2758593 | D12 | Hinggan League | Ixodes persulcatus | OR744791 | [Tick-borne encephalitis virus](https://www.ncbi.nlm.nih.gov/Taxonomy/Browser/wwwtax.cgi?id=11084) |
| BankIt2758593 | D2 | Hinggan League | Ixodes persulcatus | OR744792 | [Tick-borne encephalitis virus](https://www.ncbi.nlm.nih.gov/Taxonomy/Browser/wwwtax.cgi?id=11084) |
| BankIt2758593 | D14 | Hinggan League | Ixodes persulcatus | OR744793 | [Tick-borne encephalitis virus](https://www.ncbi.nlm.nih.gov/Taxonomy/Browser/wwwtax.cgi?id=11084) |
| BankIt2758593 | D6 | Hinggan League | Ixodes persulcatus | OR744794 | [Tick-borne encephalitis virus](https://www.ncbi.nlm.nih.gov/Taxonomy/Browser/wwwtax.cgi?id=11084) |
| BankIt2758593 | D24 | Hinggan League | Ixodes persulcatus | OR744795 | [Tick-borne encephalitis virus](https://www.ncbi.nlm.nih.gov/Taxonomy/Browser/wwwtax.cgi?id=11084) |
| BankIt2758792 | K9 | Hulun Buir | Ixodes persulcatus | OR744796 | [Onega tick phlebovirus](https://www.ncbi.nlm.nih.gov/Taxonomy/Browser/wwwtax.cgi?id=2789411) |
| BankIt2758792 | K11 | Hulun Buir | Ixodes persulcatus | OR744797 | [Onega tick phlebovirus](https://www.ncbi.nlm.nih.gov/Taxonomy/Browser/wwwtax.cgi?id=2789411) |
| BankIt2758792 | K10 | Hulun Buir | Ixodes persulcatus | OR744798 | [Onega tick phlebovirus](https://www.ncbi.nlm.nih.gov/Taxonomy/Browser/wwwtax.cgi?id=2789411) |
| BankIt2758792 | D16 | Hinggan League | Ixodes persulcatus | OR744799 | [Onega tick phlebovirus](https://www.ncbi.nlm.nih.gov/Taxonomy/Browser/wwwtax.cgi?id=2789411) |
| BankIt2758792 | D1 | Hinggan League | Ixodes persulcatus | OR744800 | [Onega tick phlebovirus](https://www.ncbi.nlm.nih.gov/Taxonomy/Browser/wwwtax.cgi?id=2789411) |
| BankIt2758792 | D12 | Hinggan League | Ixodes persulcatus | OR744801 | [Onega tick phlebovirus](https://www.ncbi.nlm.nih.gov/Taxonomy/Browser/wwwtax.cgi?id=2789411) |
| BankIt2758792 | D8 | Hinggan League | Ixodes persulcatus | OR744802 | [Onega tick phlebovirus](https://www.ncbi.nlm.nih.gov/Taxonomy/Browser/wwwtax.cgi?id=2789411) |
| BankIt2758792 | D26 | Hinggan League | Ixodes persulcatus | OR744803 | [Onega tick phlebovirus](https://www.ncbi.nlm.nih.gov/Taxonomy/Browser/wwwtax.cgi?id=2789411) |
| BankIt2758792 | D20 | Hinggan League | Ixodes persulcatus | OR744804 | [Onega tick phlebovirus](https://www.ncbi.nlm.nih.gov/Taxonomy/Browser/wwwtax.cgi?id=2789411) |
| BankIt2758792 | D13 | Hinggan League | Ixodes persulcatus | OR744805 | [Onega tick phlebovirus](https://www.ncbi.nlm.nih.gov/Taxonomy/Browser/wwwtax.cgi?id=2789411) |
| BankIt2758792 | D15 | Hinggan League | Ixodes persulcatus | OR744806 | [Onega tick phlebovirus](https://www.ncbi.nlm.nih.gov/Taxonomy/Browser/wwwtax.cgi?id=2789411) |
| BankIt2758792 | D4 | Hinggan League | Ixodes persulcatus | OR744807 | [Onega tick phlebovirus](https://www.ncbi.nlm.nih.gov/Taxonomy/Browser/wwwtax.cgi?id=2789411) |
| BankIt2758792 | D17 | Hinggan League | Ixodes persulcatus | OR744808 | [Onega tick phlebovirus](https://www.ncbi.nlm.nih.gov/Taxonomy/Browser/wwwtax.cgi?id=2789411) |
| BankIt2758792 | D6 | Hinggan League | Ixodes persulcatus | OR744809 | [Onega tick phlebovirus](https://www.ncbi.nlm.nih.gov/Taxonomy/Browser/wwwtax.cgi?id=2789411) |
| BankIt2758792 | D22 | Hinggan League | Ixodes persulcatus | OR744810 | [Onega tick phlebovirus](https://www.ncbi.nlm.nih.gov/Taxonomy/Browser/wwwtax.cgi?id=2789411) |
| BankIt2758792 | D27 | Hinggan League | Ixodes persulcatus | OR744811 | [Onega tick phlebovirus](https://www.ncbi.nlm.nih.gov/Taxonomy/Browser/wwwtax.cgi?id=2789411) |
| BankIt2758792 | D18 | Hinggan League | Ixodes persulcatus | OR744812 | [Onega tick phlebovirus](https://www.ncbi.nlm.nih.gov/Taxonomy/Browser/wwwtax.cgi?id=2789411) |
| BankIt2758792 | D11 | Hinggan League | Ixodes persulcatus | OR744813 | [Onega tick phlebovirus](https://www.ncbi.nlm.nih.gov/Taxonomy/Browser/wwwtax.cgi?id=2789411) |
| BankIt2759218 | D2 | Hinggan League | Ixodes persulcatus | OR744814 | [Tahe rhabdovirus 2](https://www.ncbi.nlm.nih.gov/Taxonomy/Browser/wwwtax.cgi?id=2983975) |
| BankIt2759218 | D3 | Hinggan League | Ixodes persulcatus | OR744815 | [Tahe rhabdovirus 2](https://www.ncbi.nlm.nih.gov/Taxonomy/Browser/wwwtax.cgi?id=2983975) |
| BankIt2759218 | D4 | Hinggan League | Ixodes persulcatus | OR744816 | [Tahe rhabdovirus 2](https://www.ncbi.nlm.nih.gov/Taxonomy/Browser/wwwtax.cgi?id=2983975) |
| BankIt2759218 | D7 | Hinggan League | Ixodes persulcatus | OR744817 | [Tahe rhabdovirus 2](https://www.ncbi.nlm.nih.gov/Taxonomy/Browser/wwwtax.cgi?id=2983975) |
| BankIt2759218 | D8 | Hinggan League | Ixodes persulcatus | OR744818 | [Tahe rhabdovirus 2](https://www.ncbi.nlm.nih.gov/Taxonomy/Browser/wwwtax.cgi?id=2983975) |
| BankIt2759662 | D1 | Hinggan League | Ixodes persulcatus | OR754420 | [Jilin luteo-like virus 2](https://www.ncbi.nlm.nih.gov/Taxonomy/Browser/wwwtax.cgi?id=2829171) |
| BankIt2759662 | D4 | Hinggan League | Ixodes persulcatus | OR754421 | [Jilin luteo-like virus 2](https://www.ncbi.nlm.nih.gov/Taxonomy/Browser/wwwtax.cgi?id=2829171) |
| BankIt2759662 | D8 | Hinggan League | Ixodes persulcatus | OR754422 | [Jilin luteo-like virus 2](https://www.ncbi.nlm.nih.gov/Taxonomy/Browser/wwwtax.cgi?id=2829171) |
| BankIt2759662 | D11 | Hinggan League | Ixodes persulcatus | OR754423 | [Jilin luteo-like virus 2](https://www.ncbi.nlm.nih.gov/Taxonomy/Browser/wwwtax.cgi?id=2829171) |
| BankIt2759662 | D16 | Hinggan League | Ixodes persulcatus | OR754424 | [Jilin luteo-like virus 2](https://www.ncbi.nlm.nih.gov/Taxonomy/Browser/wwwtax.cgi?id=2829171) |
| BankIt2759662 | D17 | Hinggan League | Ixodes persulcatus | OR754425 | [Jilin luteo-like virus 2](https://www.ncbi.nlm.nih.gov/Taxonomy/Browser/wwwtax.cgi?id=2829171) |
| BankIt2759662 | D21 | Hinggan League | Ixodes persulcatus | OR754426 | [Jilin luteo-like virus 2](https://www.ncbi.nlm.nih.gov/Taxonomy/Browser/wwwtax.cgi?id=2829171) |
| BankIt2759662 | D26 | Hinggan League | Ixodes persulcatus | OR754427 | [Jilin luteo-like virus 2](https://www.ncbi.nlm.nih.gov/Taxonomy/Browser/wwwtax.cgi?id=2829171) |
| BankIt2759662 | D27 | Hinggan League | Ixodes persulcatus | OR754428 | [Jilin luteo-like virus 2](https://www.ncbi.nlm.nih.gov/Taxonomy/Browser/wwwtax.cgi?id=2829171) |
| BankIt2759907 | G1 | Hulun Buir | Haemaphysalis concinna | OR756261 | [Tahe rhabdovirus 1](https://www.ncbi.nlm.nih.gov/Taxonomy/Browser/wwwtax.cgi?id=2983974) |
| BankIt2759915 | D2 | Hinggan League | Ixodes persulcatus | OR756262 | [Ixodes scapularis associated virus 1](https://www.ncbi.nlm.nih.gov/Taxonomy/Browser/wwwtax.cgi?id=1526525) |
| BankIt2759915 | D1 | Hinggan League | Ixodes persulcatus | OR756263 | [Ixodes scapularis associated virus 1](https://www.ncbi.nlm.nih.gov/Taxonomy/Browser/wwwtax.cgi?id=1526525) |
| BankIt2759915 | D12 | Hinggan League | Ixodes persulcatus | OR756264 | [Ixodes scapularis associated virus 1](https://www.ncbi.nlm.nih.gov/Taxonomy/Browser/wwwtax.cgi?id=1526525) |
| BankIt2759915 | D25 | Hinggan League | Ixodes persulcatus | OR756265 | [Ixodes scapularis associated virus 1](https://www.ncbi.nlm.nih.gov/Taxonomy/Browser/wwwtax.cgi?id=1526525) |
| BankIt2759915 | D21 | Hinggan League | Ixodes persulcatus | OR756266 | [Ixodes scapularis associated virus 1](https://www.ncbi.nlm.nih.gov/Taxonomy/Browser/wwwtax.cgi?id=1526525) |
| BankIt2759925 | K5 | Hulun Buir | Ixodes persulcatus | OR756267 | [Yezo virus](https://www.ncbi.nlm.nih.gov/Taxonomy/Browser/wwwtax.cgi?id=2825847) |
| BankIt2759990 | D21 | Hinggan League | Ixodes persulcatus | OR756268 | [Jilin partiti-like virus 1](https://www.ncbi.nlm.nih.gov/Taxonomy/Browser/wwwtax.cgi?id=2829172) |
| BankIt2759990 | D6 | Hinggan League | Ixodes persulcatus | OR756269 | [Jilin partiti-like virus 1](https://www.ncbi.nlm.nih.gov/Taxonomy/Browser/wwwtax.cgi?id=2829172) |
| BankIt2759990 | D1 | Hinggan League | Ixodes persulcatus | OR756270 | [Jilin partiti-like virus 1](https://www.ncbi.nlm.nih.gov/Taxonomy/Browser/wwwtax.cgi?id=2829172) |
| BankIt2762119 | B21 | Bayan Nur | Dermacentor nuttalli | OR785045 | Totiviridae sp. |
| BankIt2762121 | E5 | Bayan Nur | Hyalomma marginatum | OR785046 | Bole tick virus 3 |
| BankIt2762121 | E6 | Bayan Nur | Hyalomma marginatum | OR785047 | Bole tick virus 3 |
| BankIt2762153 | D25 | Hinggan League | Ixodes persulcatus | OR785048 | Taiga tick nigecruvirus |
| BankIt2762153 | D19 | Hinggan League | Ixodes persulcatus | OR785049 | Taiga tick nigecruvirus |
| BankIt2762153 | D17 | Hinggan League | Ixodes persulcatus | OR785050 | Taiga tick nigecruvirus |
| BankIt2762153 | D2 | Hinggan League | Ixodes persulcatus | OR785051 | Taiga tick nigecruvirus |
| BankIt2762153 | D23 | Hinggan League | Ixodes persulcatus | OR785052 | Taiga tick nigecruvirus |
| BankIt2762578 | B19 | Bayan Nur | Dermacentor nuttalli | OR785053 | Xinjiang tick associated virus 1 |
| BankIt2762580 | K13 | Hulun Buir | Ixodes persulcatus | OR785054 | Mukawa virus |
| BankIt2762582 | D2 | Hinggan League | Ixodes persulcatus | OR785055 | Jilin luteo-like virus 2 |
| BankIt2773636 | E48 | Bayan Nur | Hyalomma marginatum | OR911899 | Volzhskoe tick virus |
| BankIt2773636 | E50 | Bayan Nur | Hyalomma marginatum | OR911900 | Volzhskoe tick virus |
| BankIt2773636 | E7 | Bayan Nur | Hyalomma marginatum | OR911901 | Volzhskoe tick virus |
| BankIt2773636 | E12 | Bayan Nur | Hyalomma marginatum | OR911902 | Volzhskoe tick virus |
| BankIt2773636 | E28 | Bayan Nur | Hyalomma marginatum | OR911903 | Volzhskoe tick virus |
| BankIt2773636 | E29 | Bayan Nur | Hyalomma marginatum | OR911904 | Volzhskoe tick virus |
| BankIt2773636 | E31 | Bayan Nur | Hyalomma marginatum | OR911905 | Volzhskoe tick virus |
| BankIt2773636 | E35 | Bayan Nur | Hyalomma marginatum | OR911906 | Volzhskoe tick virus |
| BankIt2773636 | E36 | Bayan Nur | Hyalomma marginatum | OR911907 | Volzhskoe tick virus |
| BankIt2773636 | E37 | Bayan Nur | Hyalomma marginatum | OR911908 | Volzhskoe tick virus |
| BankIt2773636 | E38 | Bayan Nur | Hyalomma marginatum | OR911909 | Volzhskoe tick virus |
| BankIt2773636 | E39 | Bayan Nur | Hyalomma marginatum | OR911910 | Volzhskoe tick virus |
| BankIt2773636 | E41 | Bayan Nur | Hyalomma marginatum | OR911911 | Volzhskoe tick virus |
| BankIt2773636 | E45 | Bayan Nur | Hyalomma marginatum | OR911912 | Volzhskoe tick virus |
