# Supplementary material for: Metatranscriptomic analysis reveals the diversity of RNA viruses in ticks in Inner Mongolia, China
Source: PLoS Negl Trop Dis. 2024 Dec 11;18(12):e0012706. doi: 10.1371/journal.pntd.0012706 (PMC11634002; doi:10.1371/journal.pntd.0012706)
Supplement: S1 File — (ZIP) [file pntd.0012706.s005.zip › S1 File/Best-fit model for phylogenetic trees.docx]

| **Classification** | **Best-fit model** |
| --- | --- |
| Bunyavirales | LG+F+R10 |
| Flaviviridae | LG+F+R7 |
| Nairoviridae | LG+F+G4 |
| Chuviridae | LG+I+G4 |
| Partitiviridae | LG+F+I+G4 |
| Phenuiviridae | LG+F+R10 |
| Rhabdoviridae | LG+F+R10 |
| Solemoviridae | LG+F+R5 |
| Totiviridae | LG+F+I+G4 |

**Best-fit model for phylogenetic trees**
